# Candidate resistance genes to foliar phylloxera identified at *Rdv3* of hybrid grape

**DOI:** 10.1093/hr/uhac027

**Published:** 2022-02-20

**Authors:** Lu Yin, Avinash Karn, Lance Cadle-Davidson, Cheng Zou, Jason Londo, Qi Sun, Matthew D Clark

**Affiliations:** Department of Horticultural Science, University of Minnesota, Twin Cities, Minnesota 55018, USA; School of Life Science, Arizona State University, Tempe, Arizona 85281, USA; AgReliant Genetics LLC, Lebanon, Indiana 46052, USA; School of Integrative Plant Sciences, Cornell AgriTech, Cornell University, Geneva, New York 14456, USA; School of Integrative Plant Sciences, Cornell AgriTech, Cornell University, Geneva, New York 14456, USA; Grape Genetics Research Unit, USDA-ARS, Geneva, New York 14456, USA; Institute of Biotechnology, BRC Bioinformatics Facility, Cornell University, Ithaca, New York 14853, USA; School of Integrative Plant Sciences, Cornell AgriTech, Cornell University, Geneva, New York 14456, USA; Grape Genetics Research Unit, USDA-ARS, Geneva, New York 14456, USA; Institute of Biotechnology, BRC Bioinformatics Facility, Cornell University, Ithaca, New York 14853, USA; Department of Horticultural Science, University of Minnesota, Twin Cities, Minnesota 55018, USA

**Keywords:** candidate genes, gall-forming insect, hybrid grape, genetic mapping, fine mapping, Foliar phylloxera resistance

## Abstract

The foliage of the native grape species *Vitis riparia* and certain cold-hardy hybrid grapes are particularly susceptible to the insect pest phylloxera, *Daktulosphaira vitifoliae* Fitch. A previous study using a cold-hardy hybrid grape biparental F_1_ population (N ~ 125) detected the first quantitative trait locus (QTL) for foliar resistance on chromosome 14, designated as *resistance to Daktulosphaira vitifoliae 3* (*Rdv3*). This locus spans a ~ 7-Mbp (10–20 cM) region and is too wide for effective marker-assisted selection or identification of candidate genes. Therefore, we fine mapped the QTL using a larger F_1_ population, GE1783 (N ~ 1023), and genome-wide rhAmpSeq haplotype markers. Through three selective phenotyping experiments replicated in the greenhouse, we screened 184 potential recombinants of GE1783 using a 0 to 7 severity rating scale among other phylloxera severity traits. A 500-kb fine mapped region at 4.8 Mbp on chromosome 14 was identified. The tightly linked rhAmpSeq marker 14_4 805 213 and flanking markers can be used for future marker-assisted breeding. This region contains 36 candidate genes with predicted functions in disease resistance (R genes and *Bonzai* genes) and gall formation (bifunctional 3-dehydroquinate dehydratase/shikimate dehydrogenase). Disease resistance genes suggest a traditional R-gene-mediated resistance mechanism often accompanied by a hypersensitive response, which has been widely studied in the plant pathology field. A novel resistance mechanism, non-responsiveness to phylloxera gall formation is proposed as a function of the bifunctional dehydratase gene, which plays a role in gallic acid biosynthesis and is important in gall formation. This study has implications for improvement of foliar phylloxera resistance in cold-hardy hybrid germplasm and is a starting place to understand the mechanism of resistance in crops to gall-forming insects.

## Introduction

Grape phylloxera, *Daktulosphaira vitifoliae* Fitch (Hemiptera: Phylloxeridae), is native to North America and infests both the foliage and roots of *Vitis*, also referred to as gallicoles and radicicoles, respectively. The root form of phylloxera has been better studied than the foliar form because of outbreaks on *Vitis vinifera* in the 19^th^ century [1]. Yet, the less studied gallicoles are a problem on the native grape species *Vitis riparia* and certain increasingly planted cold-hardy hybrid varieties such as ‘Frontenac’ and ‘La Crescent’ [2]. It has been shown that foliar phylloxera infestation reduced yield of ‘Frontenac’ at Minnesota vineyards [3]. Two other studies have also shown a reduction in grape yield due to foliar phylloxera infestations on ‘Villard noir’ and ‘Seyval blanc’ [4,5]. There also seemed to be some effects of phylloxera on photosynthesis and berry soluble solid content [3,5–7]. Currently, there is no economic threshold established and phylloxera has the capability of both sexual and asexual reproduction, leading to the build-up of large populations quickly while allowing for genetic recombination [2].

Chemical control is the main management method for foliar infestations currently [2]. Chemical control can pose potential environmental and health concerns, and its use often has uneven and temporary controls prone to degradation. There are concerns that reliance on a single management method where heavy selection pressure would accelerate the development of insecticide-resistant phylloxera genotypes [2]. Phylloxera genotypes are fast evolving. A recent study reported 203 unique phylloxera genotypes, and foliar phylloxera appeared in formerly resistant scions (*V. vinifera*) in commercial European vineyards [8]. Breeding for phylloxera resistant grape varieties provides another management option contributing to the sustainability of grape production worldwide as pest populations evolve. A number of varieties like ‘Louise Swenson’, ‘Brianna’, ‘Edelweiss’ and varieties with *Vitis labrusca* linages that are actively involved in cold-hardy hybrid grape breeding programs are sources of resistance [2]. *V. vinifera*, though susceptible to root infestations, is usually a non-host for foliar phylloxera [9].

The most commonly observed and studied resistance mechanism results from the presence of both a resistance (R) gene in the plant and an avirulence gene in the insect. This gene-for-gene model was seen in the case of wheat resistance to Hessian fly and rice resistance to a gall midge where most of these R genes are single, dominant genes [10,11]. Each R gene recognizes a specific effector coded by an avirulence gene, resulting in effector-trigger immunity [12]. Effector-trigger immunity (ETI) is an accelerated resistance response and is usually accompanied by a hypersensitive response [13,14]. A hypersensitive response (HR), a well-studied resistance response in the field of plant pathology, is characterized as a necrotic response due to programmed cell death of the attacked tissue, production of toxic compounds (autofluorescent), and a disruption in the oxidative balance [15,16]. In plant-herbivorous insect systems, HR can be elicited by insect oviposition [17]. The first report of HR to insects was in grape roots to phylloxera [18]. Raman et al. has observed HR on leaves of a resistant *V. vinifera* variety upon phylloxera infestation [19].

The current literature studying gall-forming insect resistance genes in woody plants is limited. The only genetic study for resistance to a gall-forming insect in woody plants is in willow [20]. The researchers detected a major QTL for resistance to a gall midge in both years of their study and suggested that the resistance gene, instead of playing an active defense role like HR, might be a non-functional copy of the gene inducive to gall formation (hereafter referred to as “non-responsiveness”). In their QTL region, Höglund et al. did not find any nucleotide binding site leucine-rich repeat R genes (NBS-LRR) that typically trigger HR [20]. Grape phylloxera gall formation was shown to reprogram the plant leaves for nutritive tissue production similar to that of carpel formation and secondary metabolism alteration such as the up-regulation of genes in shikimate and phenylpropanoid biosynthesis [6,21]. Granett et al. observed resistance responses on roots including lower reproductive rates, lower rates of increase, and lower rates of 1^st^ instar establishment [22].

The majority of literature studying grape phylloxera genetic resistance is limited to radicicoles. Using hybrids of *V. riparia* and *V. cinerea*, a root phylloxera resistance QTL encompassing *Rdv1* was identified and characterized on chromosome 13 [23,24]. Nineteen candidate genes were identified including 6 clustered NBS-LRR genes covering the *Rdv1* locus using the *V. cinerea* haplotype [23]. Using hybrids of *V. cinerea* and *V. vinifera*, another locus for resistance to root infestation *Rdv2* was identified on chromosome 14 [25].

Using a cold-hardy hybrid grape population GE1025 (N = ~125; MN1264 × MN1246) and genotype-by-sequencing SNP markers, the first resistant QTL to foliar phylloxera resistance was identified on chromosome 14 [9]. The QTL identified with the most significantly associated marker at 5 Mbp has recently been designated as *resistance to Daktulosphaira vitifoliae locus 3* (*Rdv3*) [26]. The largest effect QTLs for foliar phylloxera traits were detected on the maternal parent, MN1264 [9]. The population was chosen because of its multiple-species ancestry including *V. vinifera, V. riparia, V. rupestris, V. labrusca, V.aestivalis* and *V. berlandieri*, some of which contribute to winter hardiness, fruit composition traits, and disease resistance [9,27]. However, the foliar resistance region is relatively wide, spanning a 10–20 cM (~7 Mbp) region, which limits efficient marker-assisted breeding and the ability to identify candidate resistance genes for the biological understanding. Selective phenotyping of informative recombinants in a large population has been proven a cost-effective way to improve mapping resolutions [28]. This strategy has been used successfully to fine map disease traits in cereal crops like barley and horticultural crops like tomato with a population size of 500–1100 [29,30]. This selective phenotyping strategy would be especially useful in woody plant species like grape because of the time, labor and space needed to establish such a large population.

Given the advantages of inter-specific hybrid grape breeding, there remains a high level of structural diversity in the genus *Vitis*. Zou et al. (2020) has thus developed the rhAmpSeq marker system that is transferable across the *Vitis* core genome [31]. This RNase H2 enzyme-dependent amplicon sequencing (rhAmpSeq) system uses the next generation sequencing platform Illumina with multi-allelic haplotype markers designed to capture SNPs and/or Indels across the *Vitis* core genome [31]. The *Vitis* core genome was developed by whole genome alignment of 10 independent de novo assemblies, while the haplotype marker approach allows multiple alleles in the germplasm to be captured instead of the commonly used biallelic SNP marker approach [31].

The aim of this study was to fine map the previously reported QTL on chromosome 14 underlying resistance to foliar phylloxera using a larger population and the rhAmpSeq approach to 1) identify closely linked markers for more effective marker-assisted breeding and 2) identify candidate genes within the region for a better understanding of resistance mechanisms.

## Results

**Figure 1:**
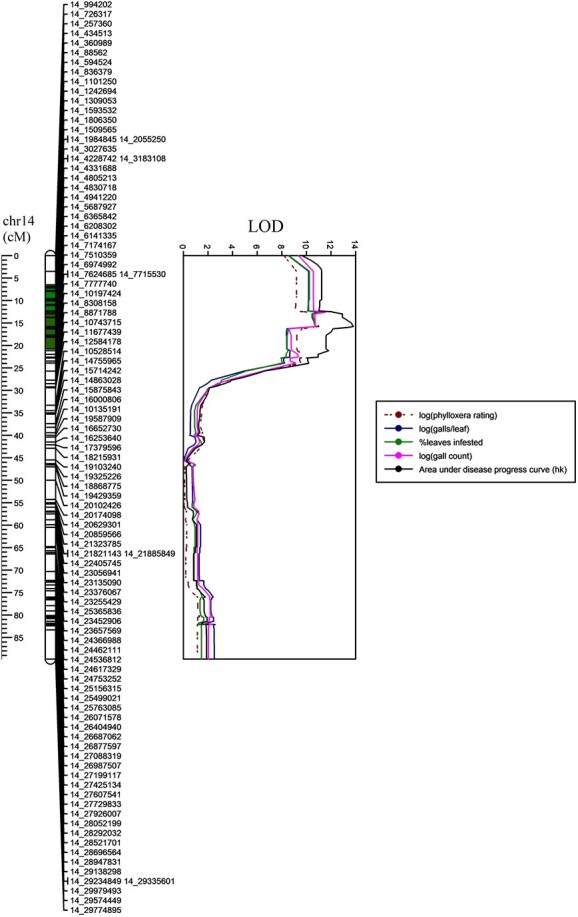
**LOD plots of phylloxera traits obtained from interval mapping in R/qtl across the genetic and physical positions on chromosome 14 of 108 selected individuals from a cold-hardy hybrid grape population, GE1783.** Green colored segment on chromosome: previously found phylloxera QTL (Clark et al. 2018).

**Figure 2:**
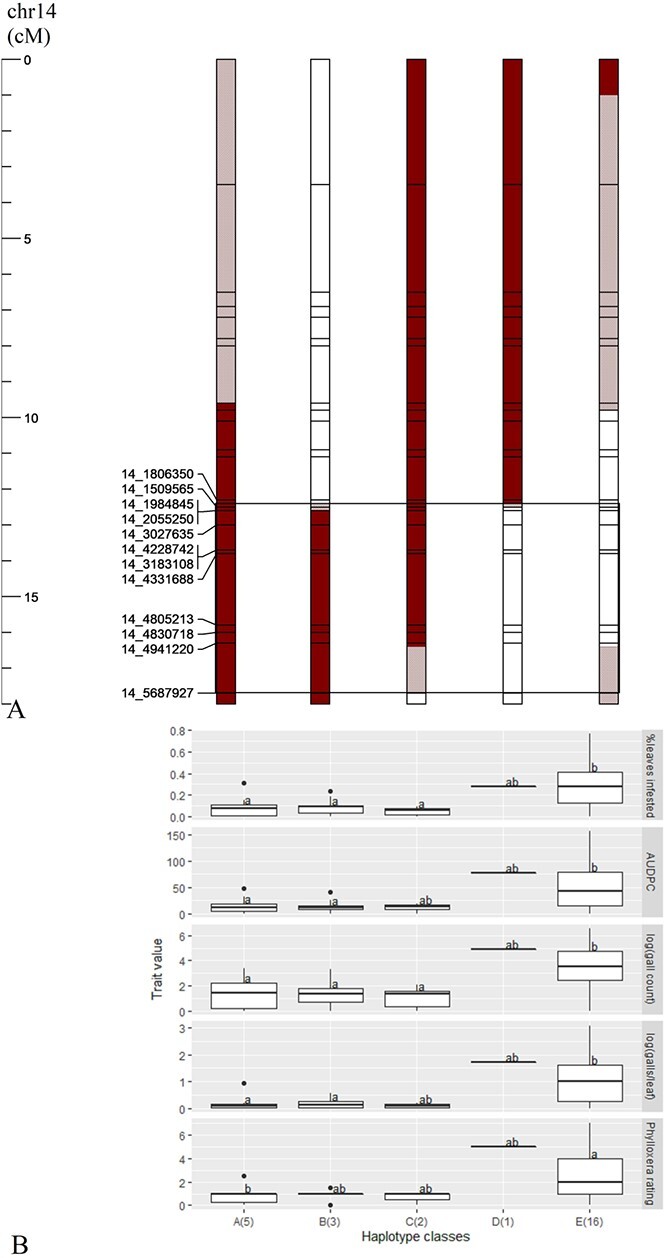
**Graphical genotypes (A) and phylloxera phenotypes (B) of five different recombinant classes at 0–18.0 cM (0–6.4 Mbp) on chromosome 14 of a cold-hardy hybrid grape population, GE1783. A** Red segments: resistant (R) phylloxera haplotype (inherited from MN1264); red-cross patterned segments: variable recombination of R haplotype; black lined box: the fine mapped region; marker name 14_1 806 350, for example, refers to haplotype marker on chromosome 14 starting at 1806350 bp. **B** Haplotype classes A to E (number of individuals per class). Lower-case letters within a trait indicate significant differences among haplotype classes (Tukey’s HSD) fitting a mixed linear model.

**Figure 3:**
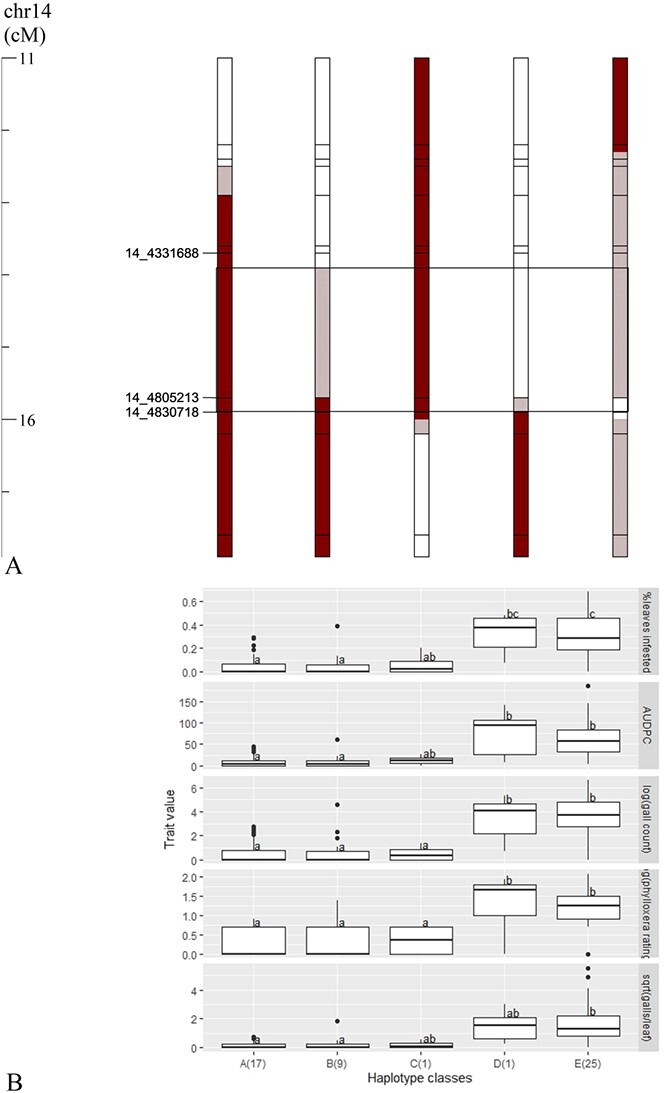
**Graphical genotypes (A) and phylloxera phenotypes (B) of five different haplotype classes at 11.1–18.0 cM (1.5–6.4 Mbp) on chromosome 14 of a cold-hardy hybrid grape population, GE1783. A** Red segments: resistant (R) phylloxera haplotype (inherited from MN1264); red-cross patterned segments: variable recombination of R haplotype; black lined box: the fine mapped region; marker name 14_4 805 213, for example, refers to haplotype marker on chromosome 14 starting at 4805213 bp. **B** Haplotype classes A to E (number of individuals per class). Lower-case letters within a trait indicate significant differences among haplotype classes (Tukey’s HSD) fitting a mixed linear model.

The large population, GE1783 (N ~ 1023), was created with the same parents as GE1025 which was used previously to identify the major foliar resistance QTL *Rdv3* on chromosome 14 by Clark et al. [9]. The whole GE1783 population was genotyped by 1387 quality-controlled rhAmpSeq markers and a total of 184 informative individuals (recombinants identified across the chromosome 14 QTL) were phenotyped in three rounds of greenhouse experiments (see Materials & Methods). We fine mapped *Rdv3* and identified the most associated rhAmpSeq marker 14_4 805 213 (Fig. 1, 2, and 3; Supplementary Table 1). Two KASP markers (S14_1984845 and S14_4 830 718) were successfully added to increase marker density at the region. The phylloxera traits screened in all experiments showed a significant genotype or haplotype class effect in Analysis of Variance (ANOVA) (P < 0.01; Supplementary Table 2) with residuals nearly normally distributed and error variances mostly equal (Supplementary Figure 1 is an example).

In Experiment 1, we screened 108 recombinant individuals across chromosome 14 and found the peak of the QTL for phylloxera traits to be at 1.5 to 4.8 Mbp (Supplementary Table 1; Fig. 1). To select the recombinants, we color-coded (graphical genotyped) the rhAmpSeq genotyping data of GE1783 seedlings that had the same alleles as the resistant parent MN1264 (Supplementary Table 1) and identified 184 recombinants that had one or two recombination events on chromosome 14 (see Materials and Methods). Selectively phenotyped individuals showed highly inflated LOD scores and high percent phenotypic variation explained as expected by phenotyping only the recombinants (Table 1). Area under disease progress curve (AUDPC) had the narrowest QTL interval spanning 3.0 to 4.9 Mbp with its peak at 4.8 Mbp. Galls per leaf, percent leaves infested per plant, and gall count showed a slightly wider QTL interval which spanned 0.7 to 4.8 Mbp and was still narrower than that of phylloxera rating. The peaks of these three traits were at 1.5 or 2.1 Mbp (Table 1). To determine which alleles were phylloxera resistant alleles, we used *effectplot* function in *R/qtl* [32] on the significant marker for each of the traits identified here. Supplementary Fig. 2 shows the *effectplot* outputs of each phylloxera trait, where alleles BC and BD were determined as resistant alleles with the B haplotype encoding resistance inherited from MN1264. The B haplotype encodes for allele 2 at marker 14_4 805 213 in Supplementary Table 1, allele 1 at marker 14_1509565 (MN1264: 1/2, MN1246: 1/1, data not shown), and allele 2 at marker 14_2055250 (Supplementary Table 1).

**Table 1:** **Quantitative trait loci detected on chromosome 14 for foliar phylloxera traits in 108 selected recombinants of a cold-hardy hybrid grape population, GE1783**. Multiple imputation interval mapping was used except for area under disease progress curve (AUDPC) where Haley–Knott interval mapping was used

**Trait**	**Peak position (cM)**	**Peak marker**	**Peak LOD**	**Start marker**	**End marker**	**Interval (cM)** ^**a**^	**%phenotypic variation explained**
Leaves infested (%)	12.5	14_1509565	11.2	14_726317	14_4 805 213	3.5–15.8	37.9
Gall count^b^	12.6	14_2055250	11.5	14_726317	14_4 805 213	3.5–15.8	38.8
Galls/leaf^b^	12.6	14_2055250	10.9	14_726317	14_4 805 213	3.5–15.8	37.2
Phylloxera rating^b^	15.8	14_4 805 213	11.0	14_257360	14_10197424	6.5–23.9	37.5
AUDPC	15.8	14 4 805 213	13.8	14 3027635	14 4941220	13.0–16.3	44.8

a based on bayesian interval

b natural log transformed

In Experiment 2, we screened 27 recombinants across 0–6.4 Mbp and we confirmed the QTL to be at 1.5 to 5.7 Mbp region (10 markers) by organizing the recombinant individuals into 15 representative haplotype classes with 1 to 4 individuals per class (Supplementary Fig. 3). For discussion purposes, we organized them into five major haplotype classes with 1 to 16 individuals per class (Fig. 2). These five major, representative classes contained differential introgressions at the target region. For example, if any of the 15 haplotype classes had the same recombination points across the target region but had different recombination points outside this region, they were grouped together. The mixed linear model found that haplotype classes A, B, and C had significantly lower phylloxera severity than classes D and E for two of the five traits (except no statistical significance was found for class D due to small sample size) (Fig. 2). Presence of the resistant allele at the markers at this 1.5 to 5.7 Mbp region (or 2.1–4.9 Mbp more conservatively speaking) resulted in a 79% reduction in percent leaves infested per plant. The average percent leaves infested of haplotype classes A, B and C was 7%, whereas the average of classes D and E was 34% such that (34%–7%)/34% = 79%.

In Experiment 3, we screened 53 recombinants across 1.5–6.4 Mbp and further narrowed the QTL to a 500-kb region (4331877–4 830 718 bp containing a single marker 14_4 805 213) by organizing the recombinant individuals into 10 haplotype classes with 1 to 11 individuals per class (Supplementary Fig. 4). Again, we organized them into five major, representative haplotype classes with 1 to 25 individuals per class (Fig. 3). The mixed linear model found that haplotype classes A, B, and C had significantly lower phylloxera severity than classes D and E for four of the five traits (except no statistical significance was found for class C due to small sample size) (Fig. 3). Presence of the resistant allele at the marker 14_4 805 213 resulted in an 86% reduction in percent leaves infested per plant. The average percent leaves infested for haplotype classes A, B, and C was 5%, whereas the average for classes D and E was 35%.

The single marker 14_4 805 213 found in the fine mapped region contains three alleles in the population studied with the resistance allele (allele 2) having two SNPs at 140 and 153 bp of the 164-bp amplicon (Supplementary Fig. 5). The resistant source MN1264 is heterozygous and has one copy of the resistance allele, which comes from “Seyval blanc” (Supplementary Table 1). For other rhAmpSeq marker sequences, see Supplementary Table 3.

The 500-kb fine mapped region contains a total of 36 candidate genes, of which 17 have annotations and three genes have multiple copies at this region: disease resistance proteins, protein BONZAI 3, and bifunctional 3-dehydroquinate dehydratase/shikimate dehydrogenase (chloroplastic) (bifunctional 3-DQD/SD) (Supplementary Table 4). Disease resistance genes include Vitvi14g02589, Vitvi14g02593, Vitvi14g02600, and Vitvi14g02603; *Bonzai3* genes include Vitvi14g00327, Vitvi14g02597, Vitvi14g02599, Vitvi14g00333, and Vitvi14g00334; genes encoding bifunctional 3-DQD/SD include Vitvi14g00338, Vitvi14g00339, and Vitvi14g00340 (Supplementary Table 4).

**Figure 4:**
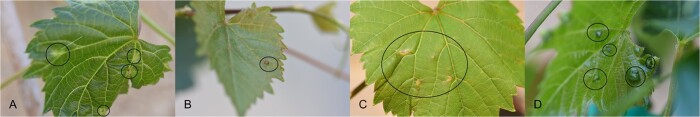
**Responses of four resistant individuals of a cold-hardy hybrid grape population, GE1783, to foliar phylloxera infestations. A** GE1783_0174, **B** GE1783_0408, **c** GE1783_0067*, **d** GE1783_0506. Circled regions are potential resistance responses. Photos were taken under Nikon D7200 camera (24.2 megapixels) with AF-S Micro Nikkor 105 mm 1:2.8G ED lens 2 weeks (*) or 5 weeks after infestation.

**Figure 5:**
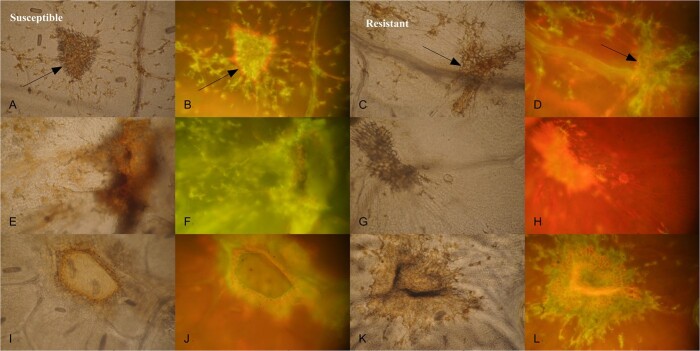
**Microscopic responses observed on leaf tissues of susceptible (A, B, E, F, I, J) and resistant (C, D, G, H, K, L) individuals of a cold-hardy hybrid grape population, GE1783.** Observations were made on greenhouse plants 4 weeks after phylloxera infestations. **A, E, I, C, G, K** Bright-field images under 200X magnification show necrotic responses. **B, F, J, D, H, L** Fluorescent images under H3 filter under the same magnification show auto-fluorescence. **A, B** GE1783_0794 (S). **E, F** MN1246 (S). **I, J** GE1783_0846 (S). **C, D, G, H, K, L** GE1783_0867 (R). Arrow points to example regions with necrotic responses and autofluorescence.

To better understand resistance gene function in the hybrid materials, whether it is NBS-LRR gene that leads to HR [19] or non-responsiveness to gall formation [20], we made additional observations on the resistance response in a few selected genotypes, macroscopically and microscopically. Potential macroscopic resistance responses include discolored spots with a necrotic center as seen with HR (Fig. 4A), incompletely formed galls with pink coloring (Fig. 4B), discolored lesions with holes (Fig. 4C), and a reduced number of galls (Fig. 4D). We also observed gall formation on some resistant genotypes. Microscopic evaluation of 10-μm thick leaf cross sections showed that the potential resistance response was collapsed parenchyma cells. We observed the collapsed cells in two of the four genotypes with the R haplotypes at the fine mapped region (Supplementary Fig. 6A). However, we also observed this type of cell in two of the seven genotypes with the S haplotypes. In addition, we observed the lignified/intact cells in five of the seven genotypes with the S haplotypes at the fine mapped region (Supplementary Fig. 6B). When we observed both a necrotic response and an autofluorescence overlay under bright-field and fluorescent microscopy, we recorded it as a resistance response [15,16] (e.g. individual GE1783_0867; Fig. 5C & D, K & L). However, we did not observe these responses consistently across all resistant haplotypes, nor were these responses only limited to the resistant genotypes. For example, we observed the resistance response on susceptible genotypes GE1783_0794 and GE1783_0846 (Fig. 5A & B, I & J), while for some resistant genotypes like GE1783_0867, the resistance response was not as obvious (Fig. 5G & H).

## Discussion

Fine mapping of the chromosome 14 foliar phylloxera resistance locus *Rdv3* provided useful markers for selection in the cold-hardy hybrid grape germplasm and identified candidate genes important for understanding resistance to gall-forming insects. Evidence suggests that we reduced the previously reported resistance QTL from a ~ 7-Mbp region down to a 500-kb region at 4.8 Mbp.

### Fine mapped region

We confirmed the effectiveness of selectively phenotyping recombinant individuals from the whole GE1783 population by the highly inflated LOD scores and high percent phenotypic variation explained in Experiment 1. The peak of the QTL for phylloxera traits was found to be at 1.5 to 4.8 Mbp, in concordance with the previous Clark et al. study [9]. AUDPC was calculated from severity ratings across five weeks after infestation, galls per leaf, percent leaves infested per plant, and gall count seemed to be the better traits to phenotype, giving narrower intervals than that of phylloxera rating. Through haplotype analysis, we confirmed the QTL to be 1.5–5.7 Mbp and further narrowed the region to a 500-kb region at 4.8 Mbp with the resistant allele at marker 14_4 805 213 reducing percent leaves infested per plant by 79% and 86%, respectively.

The fine mapped locus, *Rdv3*, with its peak at 4.8 Mbp is distinct from the root resistance locus (nodosity), *Rdv2,* with its peak at 4.2 Mbp on chromosome 14 and *Rdv1* on chromosome 13 [23–25]*.* The most likely candidate genes at *Rdv1* are NBS-LRR resistance genes [23]. *V. cinerea* has been reported to contribute resistance at *Rdv1* and *Rdv2* [25]. Since the pedigree in the current study does not contain *V. cinerea*, it is highly likely *Rdv3* is distinct from *Rdv2*. Given the complex pedigree of the population, it will require additional genotyping of ancestors in order to determine the identity by descent of the resistance gene, although preliminary evidence shows that ‘Seyval blanc’ is the likely donor, a complex French-American hybrid with *V. vinifera* ancestry, a species with known foliar resistance.

The rhAmpSeq 14_4 805 213 marker, which was found in our fine mapped region, as well as the adjacent rhAmpSeq markers from 14_2055250 to 14_6208302 which were shown to be inherited together from the ‘Seyval blanc’ (Supplementary Table 1 and 3) and KASP marker S14_4 830 718, should be useful for marker-assisted selection (including parental selection) to improve foliar phylloxera resistance in cold-hardy hybrid grapes, especially those derived from ‘Seyval blanc’. KASP markers, designed to target traditional SNPs, can be easily implemented in routine marker-assisted selection in any traditional breeding program in a cost-effective manner with basic computation skills required for rhAmpSeq. The advantages of rhAmpSeq markers are evident that they are genome-wide, multi-allelic and transferable. However, the use of such markers require highly skilled bioinformatic personnel [31].

### R-gene-mediated resistance versus non-responsiveness?

Three kinds of genes were repeatedly found in this fine mapped region that are known to be involved in disease resistance and may play a role in phylloxera resistance. Because we have not narrowed the region into a single candidate gene and cannot rule out epistatic effect, here we discuss several potential resistance mechanisms based on these three kinds of candidate genes. The first type included four (probable) disease resistance genes, all of which are presence/absence polymorphic genes, specifically one in the *Rps5* gene family which has been characterized in *Arabidopsis* [33]. Presence/absence R genes have lower nucleotide diversity in the LRR region while regular R genes evolve rapidly through the LRR region [33]. The second potential candidate gene is *Bonzai*, which is a negative regulator of R genes to a broad spectrum of pathogens and displays HR and constitutive systemic acquired resistance [34]. Liu et al. confirmed this by having mutant *Arabidopsis* plants in a *Bonzai 1/Copine 1* gene which gained resistance to a bacterial and an oomycetous pathogen and transgenic plants which looked like wildtype [34].

A third candidate gene encoding bifunctional 3-DQD/SD, catalyzes the third and fourth steps of shikimate pathway [35]. In plants, the shikimate pathway is central for the biosynthesis of aromatic amino acids which are synthesized for use as protein building blocks and the production of secondary metabolites [35]. Bifunctional enzymes are few in plants, have regulatory advantages, and have more efficient substrate conversion [36]. Bifunctional 3-DQD/SD catalyzes the dehydration of dehydroquinate to dehydroshikimate and the reversible reduction of dehydroshikimate to shikimate. Dehydroshikimate is converted to gallic acid by another enzyme dehydroshikimate dehydrogenase [37]. This process takes place in plastids but studies have also suggested a cytosolic equivalent of bifunctional 3-DQD/SD [35,37].

If the candidate resistance gene is bifunctional 3-DQD/SD, it suggests that resistance or susceptibility is related to the production of gallic acid. In other words, because gallic acid is known to be important in gall formation [38], allelic variation or loss of function at this gene leads to non-responsiveness (symptomless) to gall formation in resistance varieties. The hypothesis is that the HR-like type of response observed here (in both resistant and susceptible genotypes) could be the initial responses before gall formation to insect feeding (a wound response), instead of HR triggered by NBS-LRR genes [20]. However, this is merely a hypothesis and the exact role of gallic acid in phylloxera resistance needs to be better understood.

We observed collapsed parenchyma cells in more frequently resistance genotypes than in susceptible genotypes, which is in agreement with previous studies, where a resistant *V. vinifera* variety upon phylloxera infestations had disintegrated leaf cell membrane, vacuoles, and organelles with an elevated phenolic content at specific time points of 0–48 hours after infestation [19]. A quantitative measurement of these changes, particularly at specific time points and known feeding sites in MN1264, would aid in our understanding of the resistance response.

The range of resistance responses observed in resistant genotypes and the typical HR observed in both resistant and susceptible genotypes raise a question that whether there exists another unknown resistant mechanism to phylloxera besides traditional HR (monitored by NBS-LRR resistance genes). It could be that what we observed was not HR but a wound response before initiation of gall tissues [20]. Höglund et al. detected the co-localization of QTL of resistance to a gall midge and QTL of HR only in one of two years in willow and suggested that the role of a symptomless defense might be more important than previously thought. Before this study, Höglund et al. observed that not all resistance genotypes had HR, where a third of the resistance genotypes showed no or minor symptoms [39], similar to what we have observed here. However, before we can do such quantitative measurements, we should better characterize the resistance response in terms of its development timeline. We acknowledge the fact that it is possible the lesions we observed were not due to phylloxera, as in times it was difficult to identify the feeding-initiation sites. Looking into the resistance response by developing the grape-phylloxera system under more sterile environments would be helpful. Regarding the observation of gall formation on resistant genotypes, it will be helpful for future studies to count the living crawlers and eggs to determine if phylloxera reproductive fitness is affected.

### Implications for breeding and future directions

Our approach only considers the quantity of phylloxera galls (e.g. count, severity rating) and not the success rate of establishment or fecundity after gall formation with the exception of AUDPC which is a more representative measurement through time (narrowest QTL interval). It is unknown how successful these resistant recombinant plants were at rearing phylloxera. Studies should investigate the mother crawler fecundity and egg viability on resistant plants. The fact that we observed gall formation in some resistant genotypes is worth investigating to determine if insect population density played a role in the experiments. Granett and Kocsis evaluated gall number and crawler number including eggs, immatures and adults on different grape varieties and found that gall number corresponded to crawler number [40]. However, because of the few and non-significantly different varieties studied, they could not conclude that gall number is a good indicator of a variety’s susceptibility in place of crawler number. In experiment 2, two haplotype classes [G (4 individuals) and J (4 individuals)] might include susceptible escapes and several classes had limited replications (Supplementary Fig. 3). This might contribute to the non-significant differences observed. Large variability was observed among infested plants due to variability in plant health, fertilization, watering, and different growth conditions in the greenhouse which might have resulted in hotspots of infestation, even though re-randomization of plants was made. The LepMap3 linkage mapping approach relies on imputation, and imputation errors might have occurred due to low DNA quality of the automatic DNA extraction method used [31], which may result in the erroneous assignment of recombination events, and thus haplotype construction, in some samples.

The marker 14_4 805 213 should be investigated in the pedigree of the population to identify the source of resistance in the MN1264 background. The parents of MN1264 (MN1069 and ‘Seyval blanc’) and MN1246 (MN1200 and Frontenac) were also genotyped. Given that resistance allele comes from ‘Seyval blanc’, studies could be conducted to investigate susceptible × ‘Seyval blanc’ populations to confirm if resistance is inherited from ‘Seyval blanc’ and/or which ancestor species. For example, studies could confirm 1) if resistance is inherited from a *V. vinifera* ancestor and 2) if the locus is conserved or evolved with phylloxera biotypes. Sequence variations could be compared at the candidate genes between MN1264 and MN1246 and their parents to provide further insights of inheritance of resistance. The selective phenotyping approach, using the fine mapping population we developed, will be a useful resource for identifying candidate genes for other traits previously mapped in GE1025, including color [41], trichome density [42], cluster compactness [43], and powdery mildew resistance [27].

This study serves as a starting place to study resistance gene function to gall-forming insects and contributes to the limited existing literature. This study fine mapped the *Rdv3* locus which confers resistance to foliar phylloxera in the cold-hardy hybrid grape germplasm. The rhAmpSeq marker at this gene should be important for marker-assisted selection to improve phylloxera resistance in cold-hardy hybrid grape breeding programs. *Rdv3* is the major QTL for foliar phylloxera resistance in this germplasm and selection at this locus will utilize the resistance sources in this germplasm, providing another management method to chemical control, which is the only commonly used control method of this pest currently. The candidate genes identified, together with the resistance response observed, suggests that the mechanism underlying resistance might be more complex than previously thought. To better understand the resistance mechanism would allow for more precise breeding that leads to Integrated Pest Management.

## Materials & methods

### Plant & colony materials

The GE1783 population (N ~ 1023; MN1264 × MN1246) was created by controlled pollinations in June 2017 and is an expansion of the GE1025 population [9]. Seeds were cold stratified at ~2°C for four months and then germinated in Sungro® Propagation Mix with a thin layer of Nodampoff® sphagnum moss in the greenhouse at 24°C (watered when dried). In April 2018, seedlings were transferred to individual pots (6.4 × 6.4 × 14.0 cm) in Midwest Perlite (Appleton, WI) and M1 Professional Mix (Grower Select®) in 3:10 volume ratio with 2739 ppm 14–14-16 controlled release fertilizer with micronutrients. After several weeks of growth, tissues of the youngest expanding leaf (<1 cm diameter) of each of 1023 seedlings were collected for DNA extraction and genotyping. In May 2018, the seedlings were moved outside under shade cloth for acclimation for ~3 weeks, and then hand-planted into the nursery at the UMN Horticultural Research Center (HRC) at Excelsior, MN with a 30.5 cm spacing within rows and 122.0 cm spacing between rows. Experimental plants that were identified as recombinants at the resistant locus (Genotyping section) were collected from field cuttings, propagated, and screened for phylloxera resistance in the greenhouse (Phenotyping section). Experiment 1 was conducted on softwood cuttings of 108 nursery seedlings. The nursery vines were dug up in fall and overwintered in cold storage under moist sawdust before being hand-planted into the vineyard at the HRC in June 2019. Experiment 2 was conducted on hardwood cuttings of 27 overwintering field vines that were propagated at the end of January 2020. Experiment 3 was conducted on softwood cuttings of 53 field grown vines.

Cuttings were rooted at the Plant Growth Facility at St. Paul, MN. To propagate cuttings for Experiment 1, more than 5 reps of 1-node cuttings of each genotype with a ~ 2.5 cm diameter leaf were dipped in Hormex® rooting powder containing 0.3% indole-3-butyric acid, planted in perlite, and maintained under day-time misting (every 8 minutes for 6 seconds). After one month, rooted cuttings were transplanted into Sungrow® Professional Growing Mix (SS#8-F2) with 4.3 g/L Osmocote® Plus slow-release fertilizer [15–9–12] in 8.9 × 8.9 × 12.7 cm pots. Plants were staked regularly and watered when needed under 16-hr day HID and ~ 26°C in the greenhouse until infestation. Plants were fertilized biweekly with Peters® Professional Peat Lite Special 20–10-20. To propagate cuttings for Experiments 2 and 3, more than 8 reps of 1-node cuttings of each genotype were similarly propagated except being potted into short pots (6.4 × 6.4 × 14.0 cm) first, and when plants were over 10 cm tall, transferred to tall pots (6.4 × 6.4 × 22.9 cm) and maintained under ~20°C.

The phylloxera colony used to infest the plants was maintained on susceptible “Frontenac” and MN1246 grapevines grown in the greenhouse. The colony was started with a single gall collected in a Minnesota vineyard of a naturally infested vine. Plants were maintained under ~24°C and 16-hr supplemental lighting.

### Genotyping

Genotyping was conducted on GE1783 seedlings and parents using RNase H2 dependent amplicon sequencing (rhAmpSeq) marker platform [31]. The same rhAmpSeq genotyping data/protocol was used previously to fine map leaf trichome density in this population [42]. Briefly, collected leaf tissues were oven dried at 50°C overnight and sent to Intertek AgriTech (Sweden), where DNA was extracted using an automated magnetic bead method using the sbeadex kit by LGC (Teddington, U.K.). Two thousand of 250-bp amplicon markers that were evenly distributed across the *Vitis* core genome (average marker distance = 200 kb) were pair-end sequenced on Illumina NextSeq 500 [31] using the PN40024 v2 reference genome with v3 annotations [44]. Because multiple SNPs, insertions, and/or deletions can occur in each amplicon, these markers represent up to 4 alleles per locus in this F_1_ population of two heterozygous parents [31]. A sex-averaged consensus genetic map was constructed from 1387 quality-controlled rhAmpSeq markers in Lep-MAP3 v 0.2 [45] as previously described [31,42]. For details on how we identified haplotype variants and constructed the genetic map to obtain the 4-way phased genotype data, see Zou et al. (2020) [31].

To increase marker density for fine mapping, additional kompetitive allele specific PCR (KASP®) genotyping (LGC, Teddington, U.K.) was conducted on 83 of the recombinants (mostly phenotyped in Experiment 2 and 3). Primers were designed from GBS SNP markers (S14_1984845, S14_1984845, S14_3322049, and S14_4 830 718) previously associated with the resistance QTL on chromosome 14 [9]. Primers were designed using default parameters of the PrimerQuest tool (IDT, Coralville, Iowa) with melting temperature of 52–65°C (60°C optimal), amplicon size of 50–100 bp (50 optimal), and the last base at 3′ being alternative versions of the SNP, where allele 1 has binding site to fluorophore FAM added at the 5′, while allele 2 has binding site to fluorophore HEX added at the 5′ [46].

To identify which individuals to collect cuttings from and screen for phylloxera resistance, we selected recombinant individuals based on the rhAmpSeq genotype data across specific target regions. For Experiment 1, 180 representative recombinant individuals (≤ 5 replications/individual) across chromosome 14 were selected. To select recombinants, we looked at polymorphic markers on chromosome 14 in the whole GE1783 population and color-coded the alleles of each individual’s haplotype that were the same to the resistant parent allele. Then we selected the recombinants having 1 or 2 recombinations on chromosome 14. Due to the young vine age, 108 individuals were successfully propagated and screened. For Experiment 2, based on preliminary results, 39 recombinant individuals (≤ 8 replications/individual) across the region spanning 0 to 6.4 Mbp (on chromosome 14) were selected, where 27 individuals were successfully propagated and screened. For Experiment 3, based on preliminary results, 72 recombinant individuals (≤ 8 replications/individual) across the region spanning 1.5 to 6.4 Mbp were selected, where 53 individuals were successfully screened.

### Phenotyping

When plants were about 30-cm tall, the second mature leaf, observed as the youngest non-tender fully expanded leaf, of each plant was infested with a large gall using an alligator hair clip [9]. To ensure uniform infestation and encourage movement of crawlers among plants, plants were arranged in close proximity to each other and trays were re-randomized at two weeks after infestation (Experiment 1) or weekly for three weeks after infestation (Experiments 2 and 3).

Each plant was scored for phylloxera severity using a 0 to 7 scale [9] each week after infestation for five weeks. At four weeks after infestation, the number of galls, number of leaves, and number of infested leaves were counted per plant, where the percent leaves infested (number of infested leaves / number of leaves per plant) and the number of galls per leaf (number of galls/number of leaves) were calculated. Area under disease progress curve (AUDPC) was calculated from the severity rating across five weeks.

### Fine mapping

For Experiment 1, a larger number of recombinant individuals across a wider region on chromosome 14 were analyzed using interval mapping in *R/qtl* [47]. For Experiments 2 and 3 with smaller experiment sizes and refined regions of interest based on preliminary results, a haplotype analysis was conducted to compare the phylloxera severity traits among haplotype classes. Before either analysis, analysis of variance (ANOVA) was conducted on phylloxera traits fitting a linear model of genotype and replication effects in R version 3.6.0 [32] to confirm there was a significant genotype effect. Each model was examined for residual normality and equal error variances using the *plot.lm* function [32]. For a trait with non-normal residual distribution, square-root and natural log transformations were performed and the transformation that improved the normality was used for subsequent genetic analysis.

#### Interval mapping

Interval mapping using *scanone* function in *R/qtl* was conducted for Experiment 1 [47]. For traits or transformed traits with normal residual distributions, a multiple imputation method was performed with 256 imputations and a step size of 1. For traits with non-normal residual distributions, a Haley–Knott method was performed instead. The genome-wide 5% logarithm of odds (LOD) threshold for significant QTL was determined. The 95% Bayes credible interval was used to determine the QTL interval. To determine the resistant haplotype, allele effects were examined for each marker at the peak of a QTL using *effectplot* function and the alleles that gave the phylloxera resistant (R) or susceptible (S) phenotypes were recorded. The results obtained from *R/qtl* were visualized in MapChart [48].

#### Haplotype analysis

For Experiments 2 and 3, GE1783 individuals with different recombinations of R and S haplotypes at 0 to 6.4 Mbp on chromosome 14 were organized into unique haplotype classes. To compensate for an unbalanced design, a mixed linear model was used fitting haplotype class as a fixed effect, individuals within each class and replication as random effects using *lmer* function of the *lme4* package in R [49]. To test for a significant haplotype effect, corresponding ANOVA was also conducted. Haplotype class means were separated using Tukey’s HSD with the *glht* function in *mulcomp* package and the results were visualized using *ggplot2* in R [50,51]. Graphical genotypes of different haplotype classes were visualized in MapChart [48].

#### Identification of candidate genes

Candidate resistance genes were identified based on the physical positions of the fine mapped region from the PN40024 *12X* v2 reference genome with v3 annotations [44]. The annotations were queried by the corresponding Refseq gene IDs (e.g. LOC100252189) at NCBI.

### Additional observations of resistance responses

To better understand the resistance function, we made additional observations on a few selected genotypes. This data was not collected on all phenotyped individuals, serving as a first step to understand potential resistance responses. Macroscopically, at two or five weeks after infestation, we took notes on 5 susceptible and 10 resistant GE1783 genotypes and found responses that were potential HR responses including necrotic responses, discoloration, and pink discoloration. In addition to HR, we also took notes on other resistance responses such as incomplete gall formation and reduced number of gall formation on a few resistant genotypes such GE1783_0174, GE1783_0408, GE1783_0067, and GE1783_0506. After four weeks of infestation, we made microscopic observations on 10-μm cross sections of fixed and stained young leaf tissues of 7 susceptible and 4 resistant GE1783 genotypes. Fixed leaf tissues were stained with 1% methylene blue, sectioned with a microtone, and observed under 200X magnification (Appendix of Yin Thesis 2020). After four weeks of phylloxera infestations, leaf tissue samples of MN1246 (susceptible parent), susceptible genotypes GE1783_0794 and _0846, and a resistant genotype _0867 were cleared and fixed for microscopic observations [52]. Bright-field images and fluorescence images were taken under 200X magnification of a light microscope and in the auto-fluorescence interference blue range using the UV lamp (420–490 nm excitation filter, dichroic mirror 510 nm, barrier filter 515 nm [Ernst Leitz Wetzlar 307–143.004 microscope; Sony α5100 camera with a Leitz Wetzlar 519 749 lens; Ernst Leitz Wetzlar GmbH, Germany]). A hypersensitive response (HR) is characterized by both a necrotic response and an auto-fluorescent response [15,16].

## Acknowledgements

The authors thank James Luby and William Hutchison for their insights on gall-forming insects; Laise Moreira, Erin Treiber, Killian Harnish, Mitchell Bengtson, and Jack Olson for assistance with phenotyping; Nelson Garcia, Changbin Chen, Akhil Uppu, Brian Prior, and Benjamin Held for assistance with microscopy work; UMN Plant Growth Facility crew for providing greenhouse resources and services; John Thull, Jennifer Thull, and Colin Zumwalde for field maintenance of GE1783; Baylee Miller and Jack Tillman for KASP primer design information; Marty Anderson for assistance in KASP genotyping; Yinjie Qiu for lending the Nikon camera Micro lens. Funding was provided by USDA-NIFA Specialty Crop Research Initiative Award No. 2017-51181-26829, Minnesota Department of Agriculture Specialty Crop Grant, and UMN Grant-in-Aid.

## Author Contributions

L.Y. conducted the research and wrote the manuscript; M.C. supervised the research and edited the manuscript; A.K. constructed GE1783 linkage map, conducted rhAmpSeq genotyping, and identified recombinants for Experiment 1; L.C.D. coordinated genotyping efforts and conducted rhAmpSeq genotyping - primer design; C.Z. conducted rhAmpSeq genotyping of GE1783, parents, and grandparents; J.L. conducted rhAmpSeq genotyping - transcriptomic experiment; Q.S. conducted rhAmpSeq genotyping - exons identification.

## Data Availability

The genotypic rhAmpSeq data of GE1783 has been published in Zou et al. (2020). The phenotypic data of GE1783 recombinants are to be deposited at Data Repository of University of Minnesota.

## Conflict of interests statement

The authors declare that there is no conflict of interests in this study.

## Supplementary data

Supplementary data is available at *Horticulture Research Journal* online.

## Supplementary Material

Web_Material_uhac027
