# Supplementary material for: Candidate resistance genes to foliar phylloxera identified at Rdv3 of hybrid grape
Source: Hortic Res. 2022 Feb 20;9:uhac027. doi: 10.1093/hr/uhac027 (PMC8976690; doi:10.1093/hr/uhac027)
Supplement: Web_Material_uhac027 [file web_material_uhac027.zip › Supplementary Fig. 5.pdf]

```
1   GTCTTTTGGTGTGTATATGGCATCAAATACTAGGGAGTAATGCTTCAAAGCATGCTGCAAAAATCAAACAAGTACACAGA   80
2   GTCTTTTGGTGTGTATATGGCATCAAATACTAGGGAGTAATGCTTCAAAGCATGCTGCAAAAATCAAACAAGTACACAGA   80
3   GTCTTTTGGTGTGTATATGGCATCAAATACTAGGGAGTAATGCTTCAAAGCATGCTGCAAAAATCAAACAAGTACACAGA   80

1   AGGAAGCCTAATTATTACTTAGAAAAGTGAAAATCAATGGAGTGAATTTTATGGATGGTTACAGCATTTGGCAATGGGGC   160
2   AGGAAGCCTAATTATTACTTAGAAAAGTGAAAATCAATGGAGTGAATTTTATGGATGGCTACAGCATTTGGGAATGGGGC   160
3   AGGAAGCCTAATTATTACTTAGAAAAGCGAAAATCAATGGAGTGAATTTTATGGATGGTTACAGCATTTGGCAATGGGGC   160

1   AAAC   164
2   AAAC   164
3   AAAC   164
```
