# Supplementary figures and images for: Candidate resistance genes to foliar phylloxera identified at Rdv3 of hybrid grape
Source: Hortic Res. 2022 Feb 20;9:uhac027. doi: 10.1093/hr/uhac027 (PMC8976690; doi:10.1093/hr/uhac027)

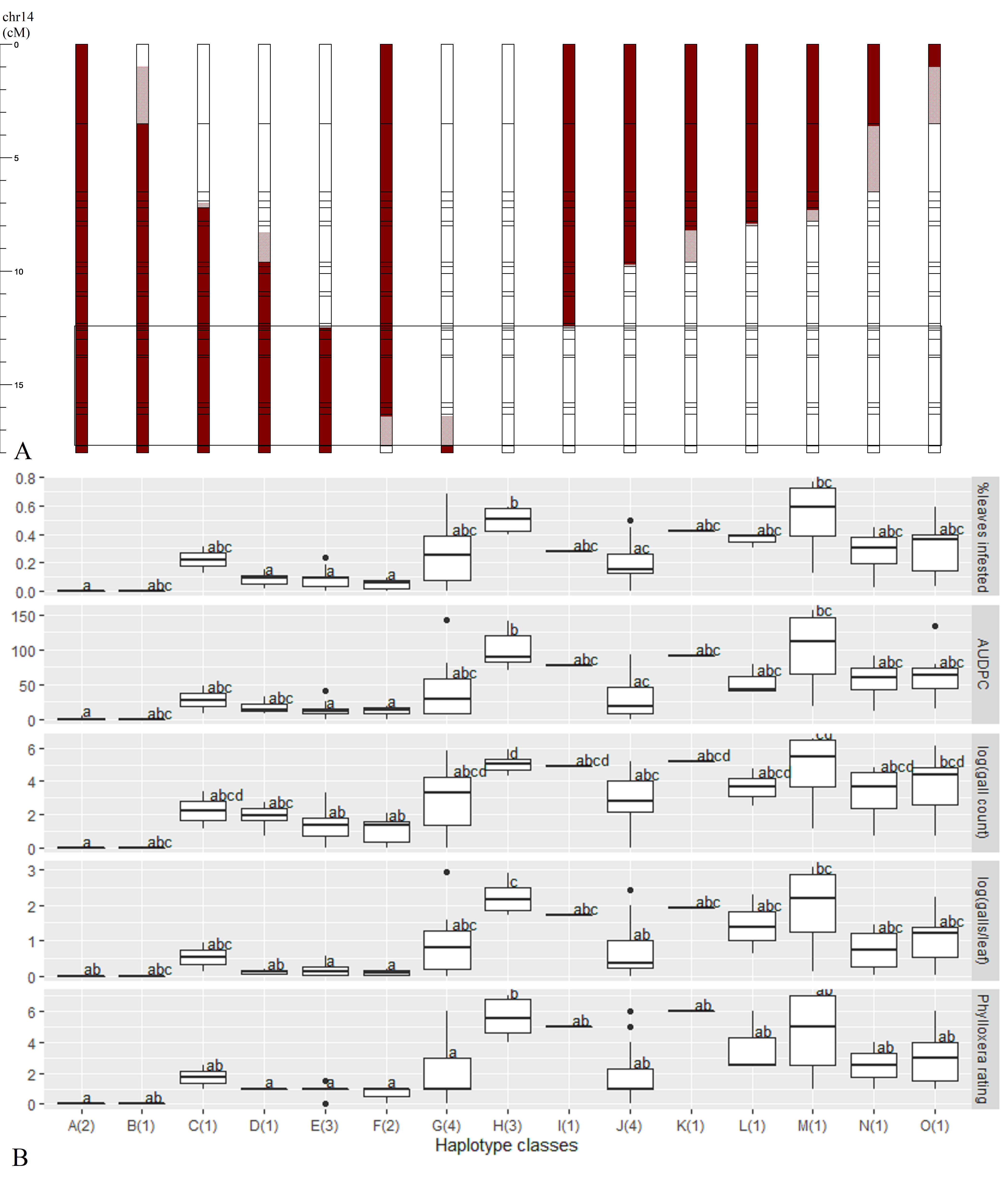

Supplement: Web_Material_uhac027 [file web_material_uhac027.zip › Supplementary Fig 3..tif]

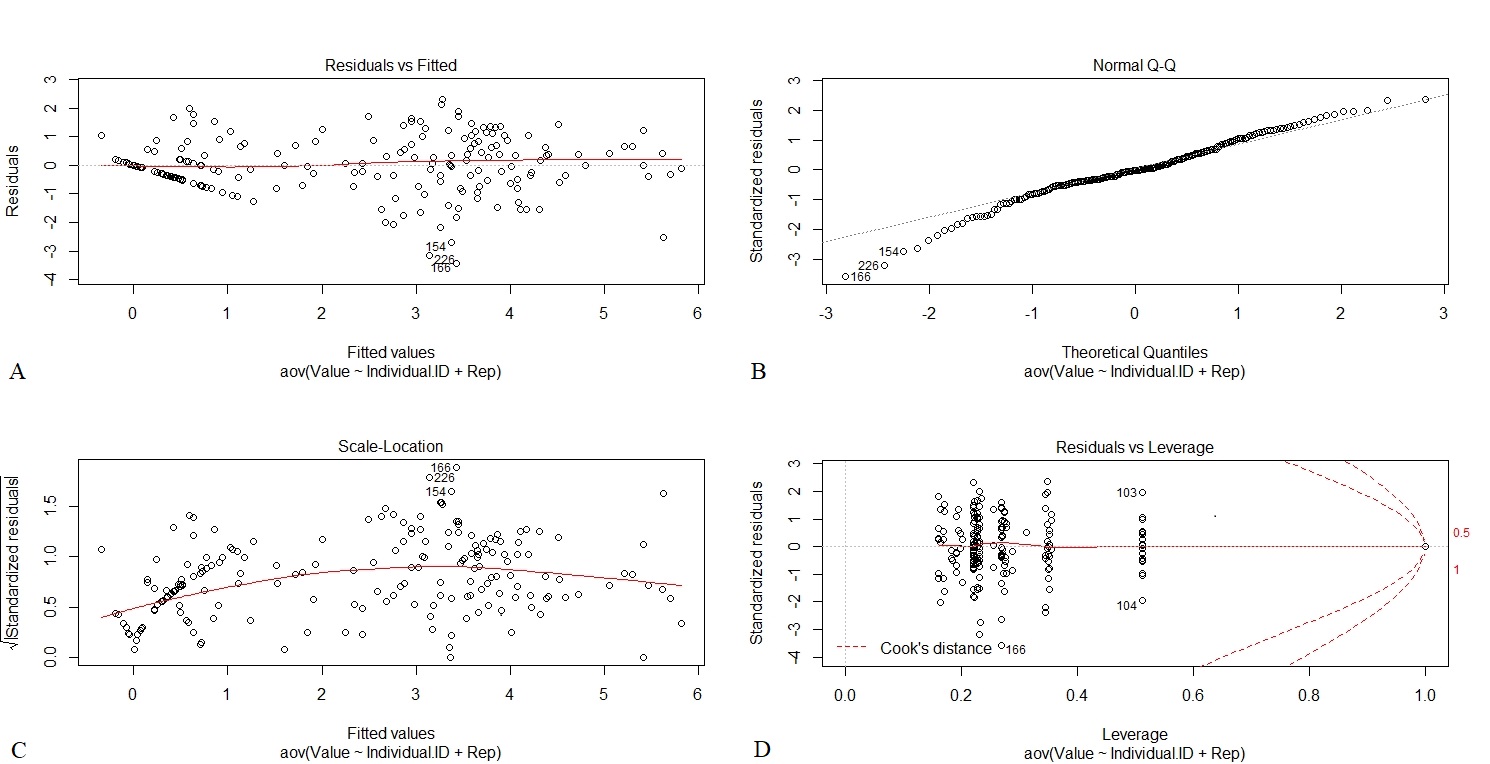

Supplement: Web_Material_uhac027 [file web_material_uhac027.zip › Supplementary Fig. 1.jpg]

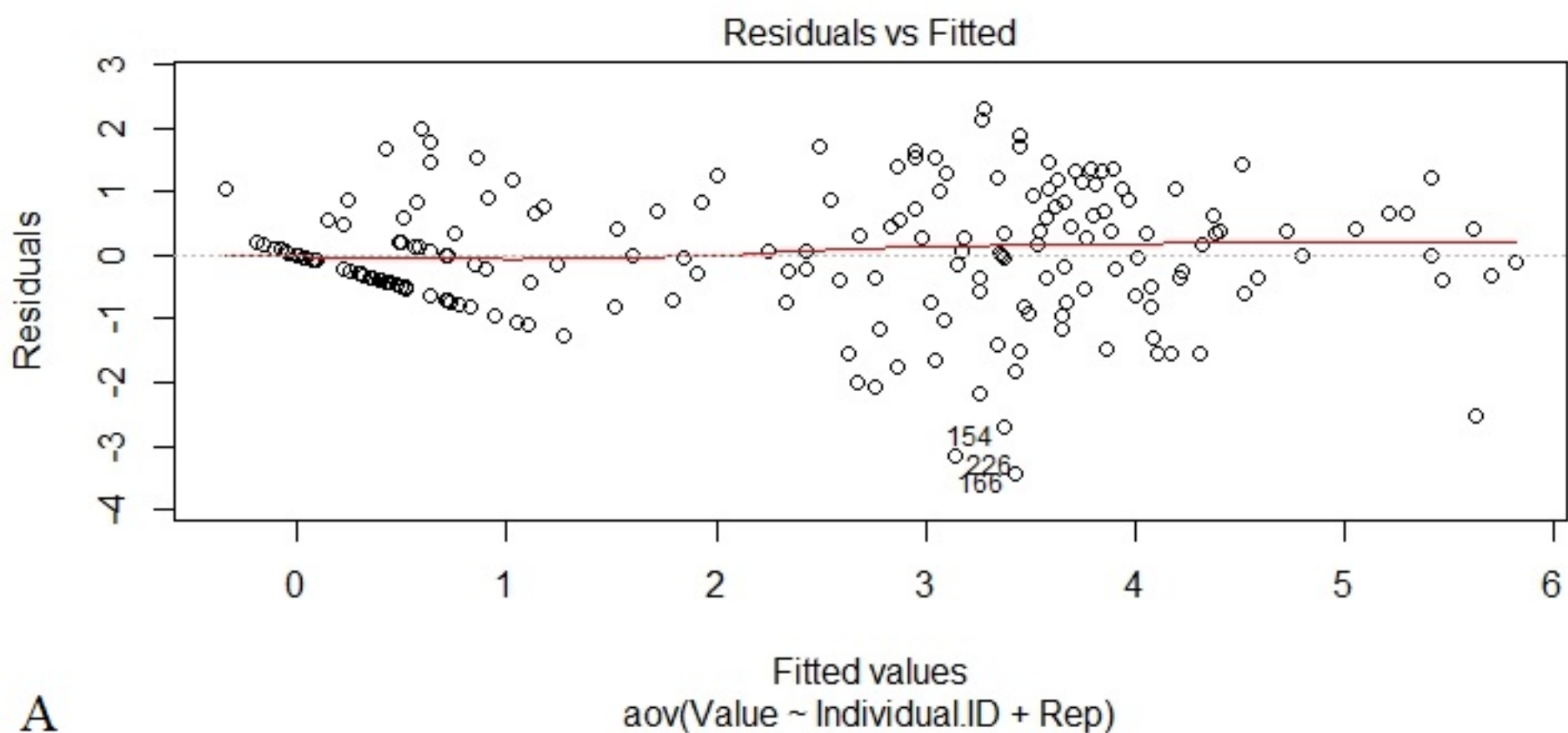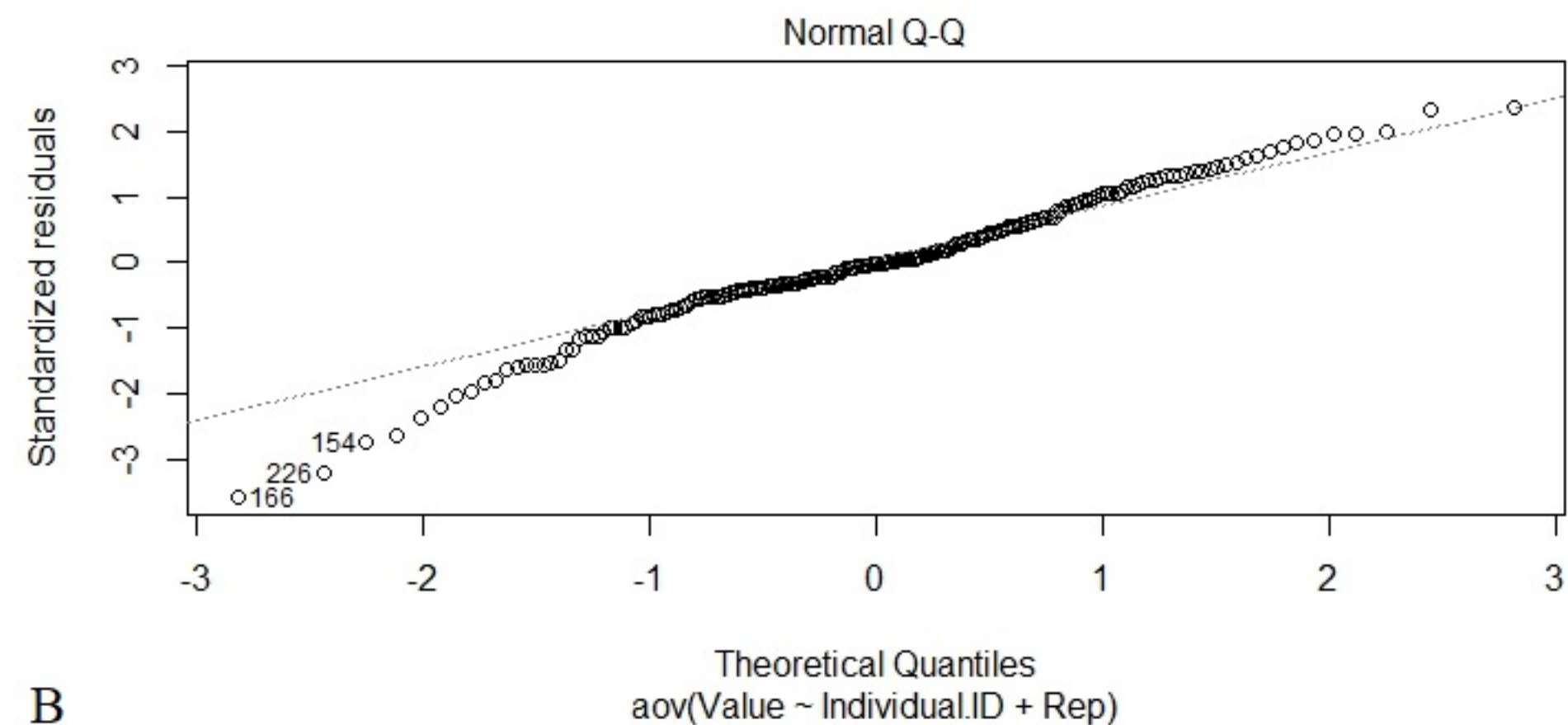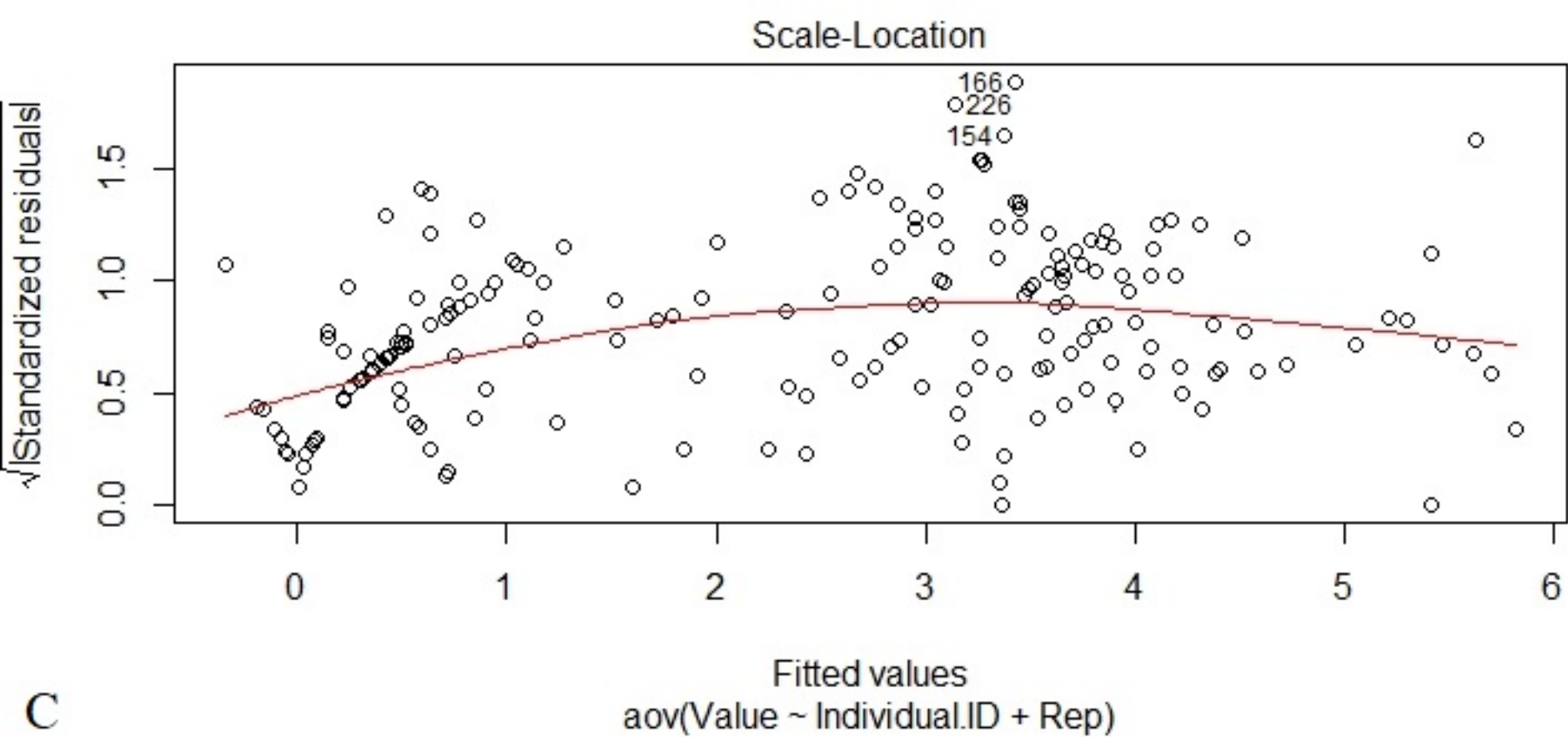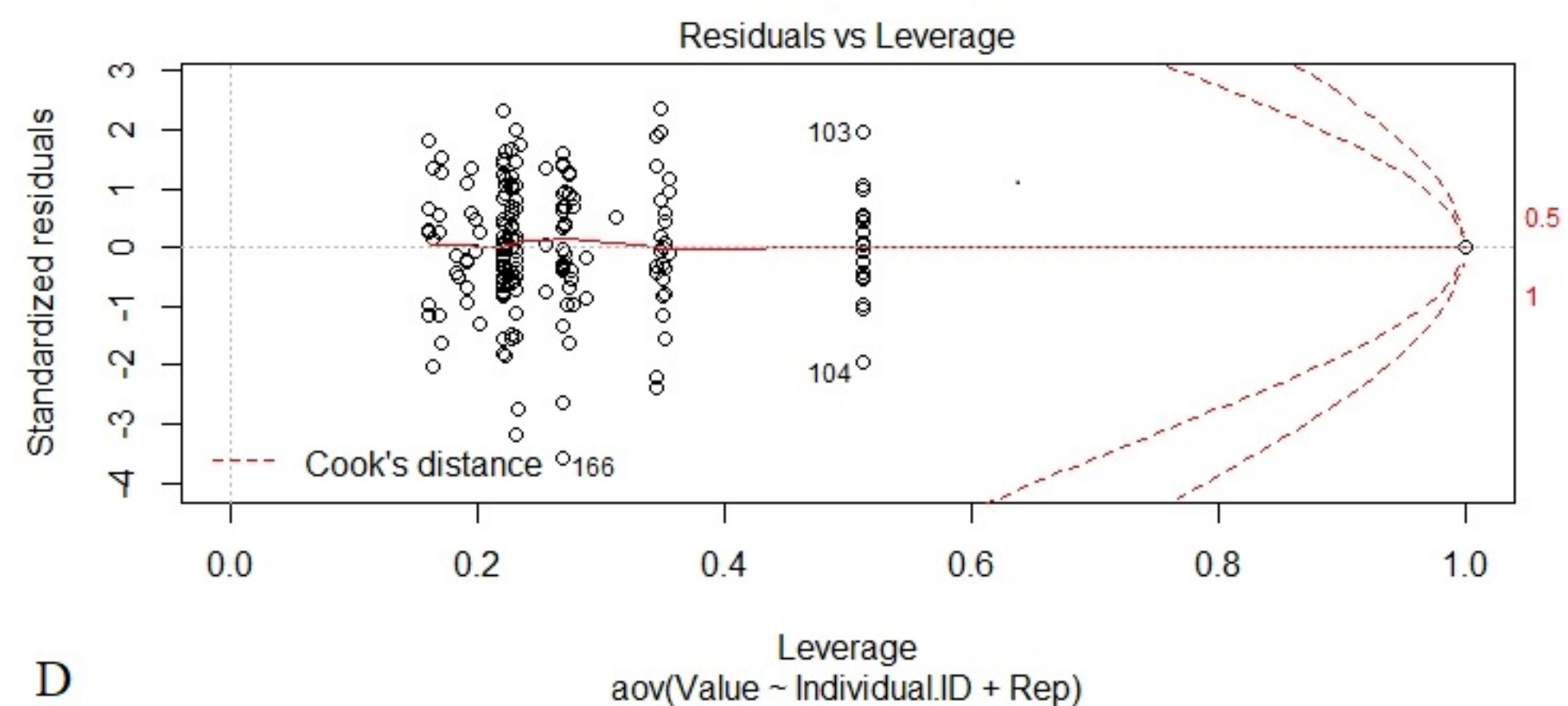

Supplement: Web_Material_uhac027 [file web_material_uhac027.zip › Supplementary Fig. 1.pdf]

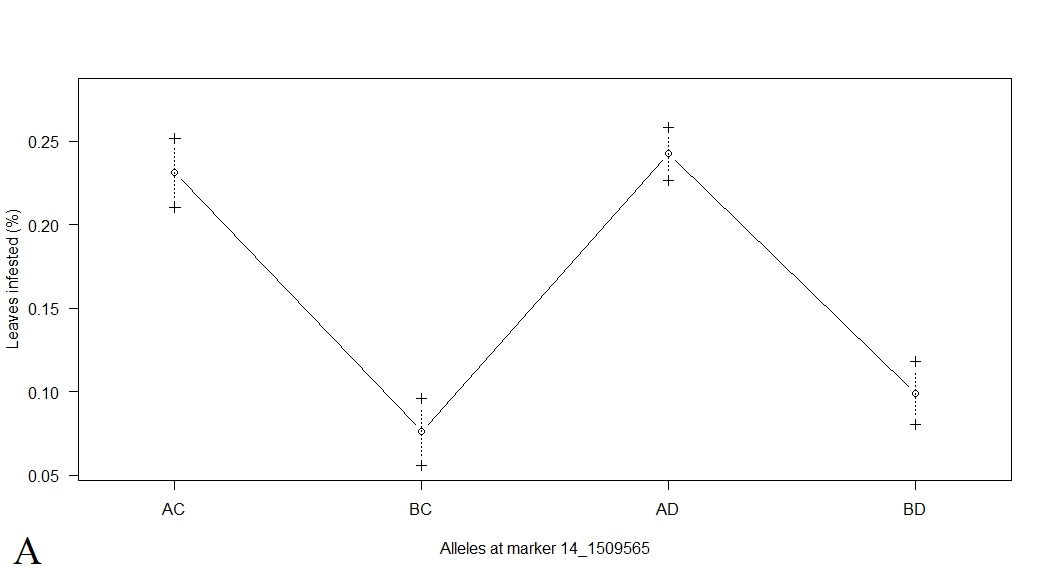

Supplement: Web_Material_uhac027 [file web_material_uhac027.zip › Supplementary Fig. 2A.jpg]

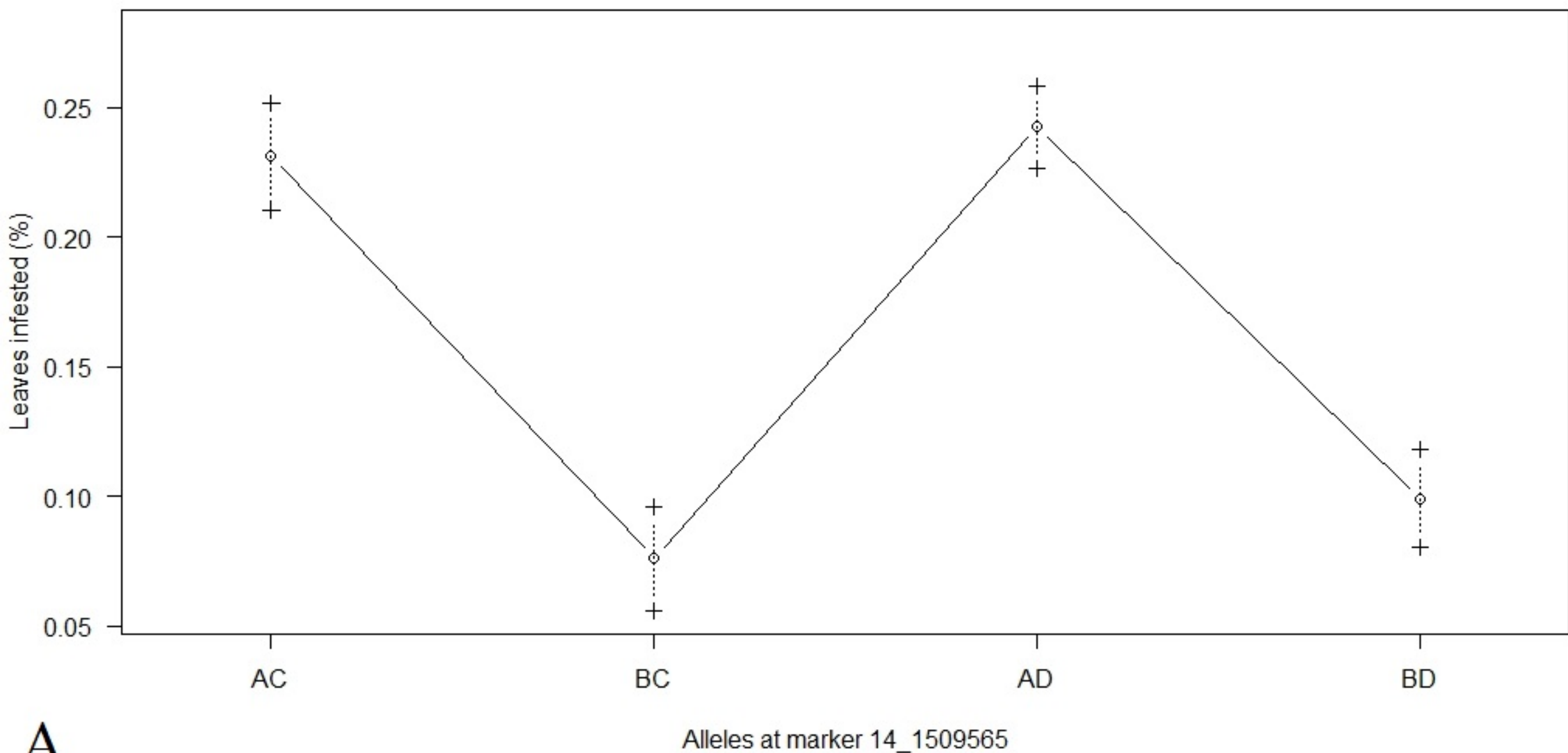

Supplement: Web_Material_uhac027 [file web_material_uhac027.zip › Supplementary Fig. 2A.pdf]

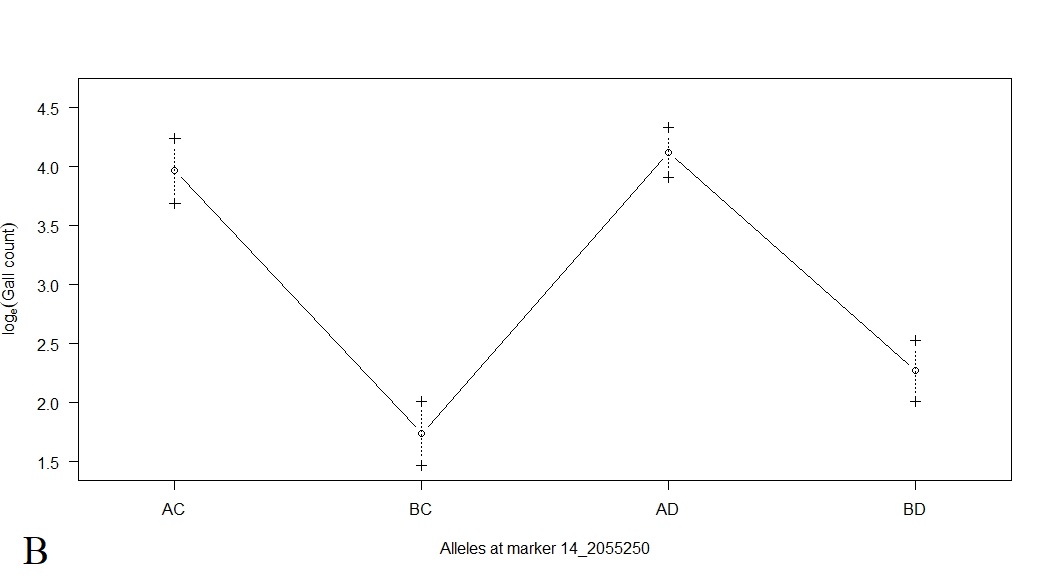

Supplement: Web_Material_uhac027 [file web_material_uhac027.zip › Supplementary Fig. 2B.jpg]

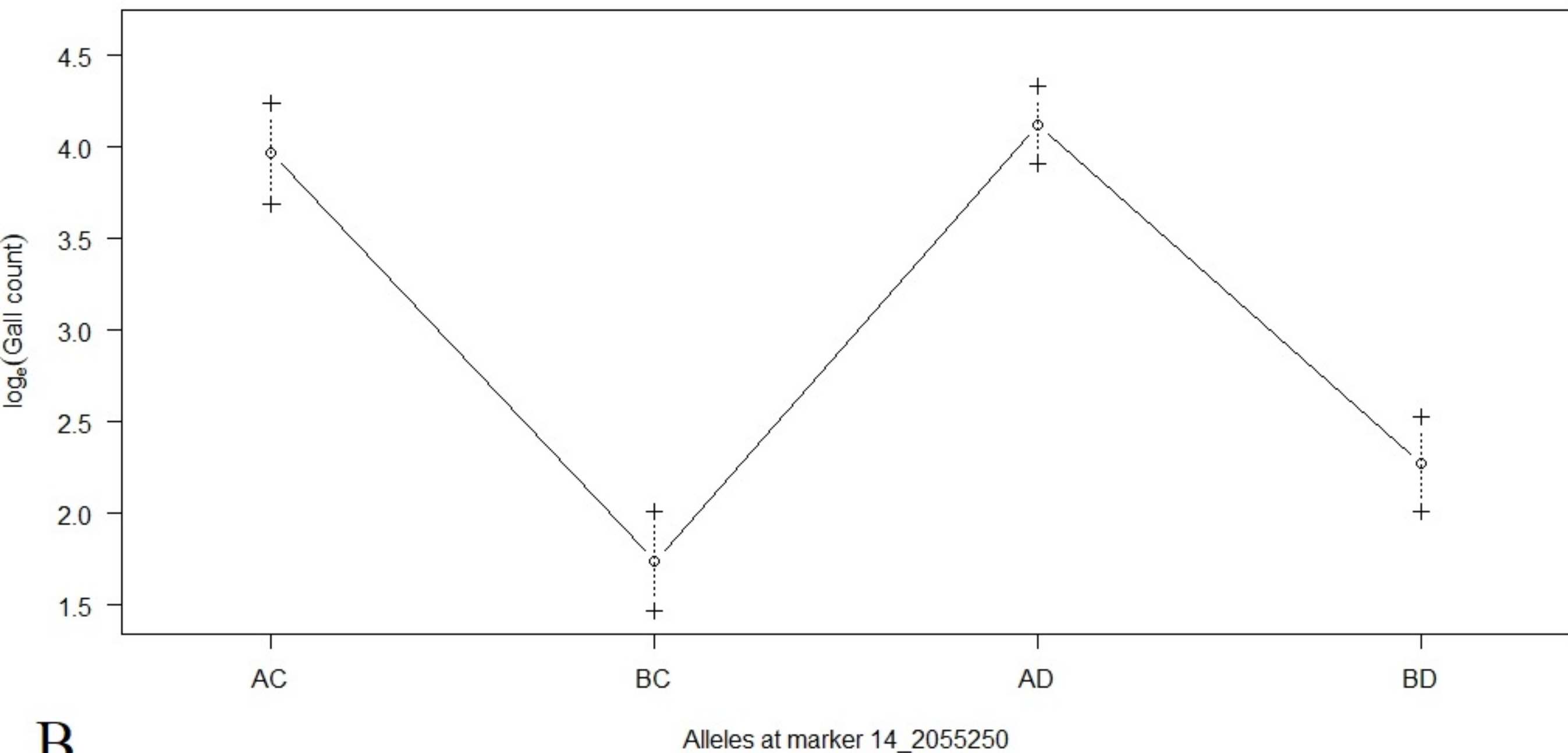

Supplement: Web_Material_uhac027 [file web_material_uhac027.zip › Supplementary Fig. 2B.pdf]

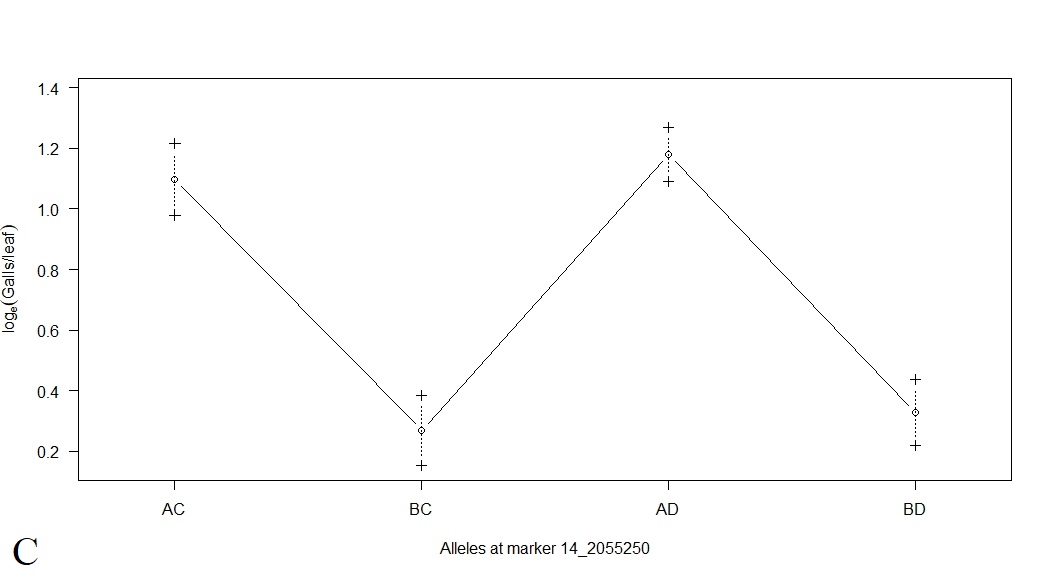

Supplement: Web_Material_uhac027 [file web_material_uhac027.zip › Supplementary Fig. 2C.jpg]

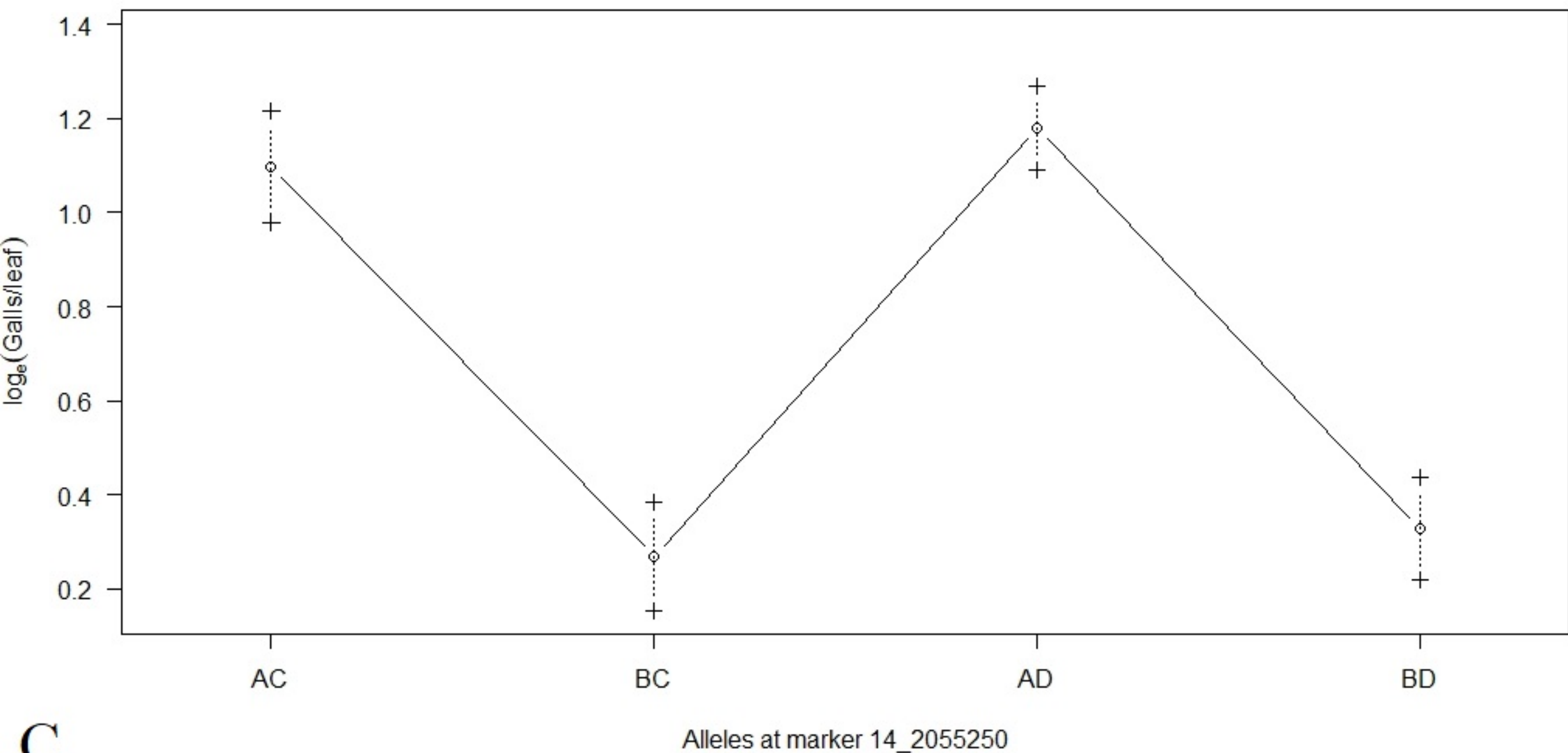

Supplement: Web_Material_uhac027 [file web_material_uhac027.zip › Supplementary Fig. 2C.pdf]

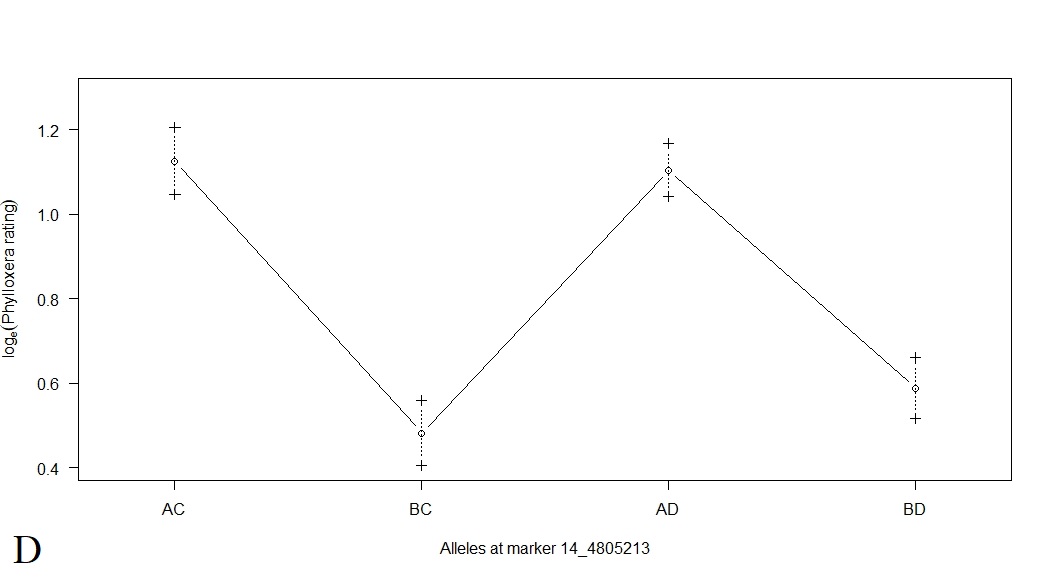

Supplement: Web_Material_uhac027 [file web_material_uhac027.zip › Supplementary Fig. 2D.jpg]

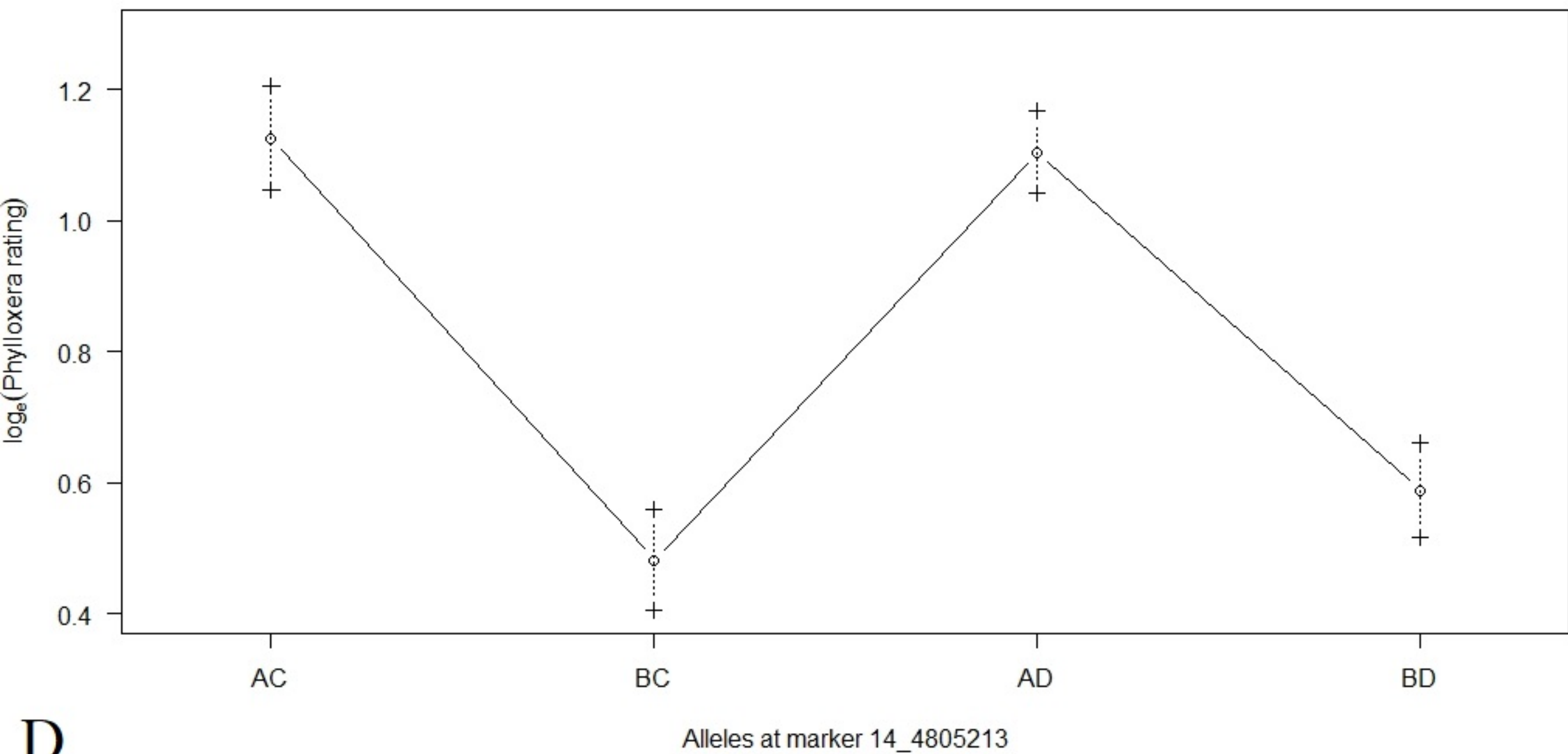

Supplement: Web_Material_uhac027 [file web_material_uhac027.zip › Supplementary Fig. 2D.pdf]

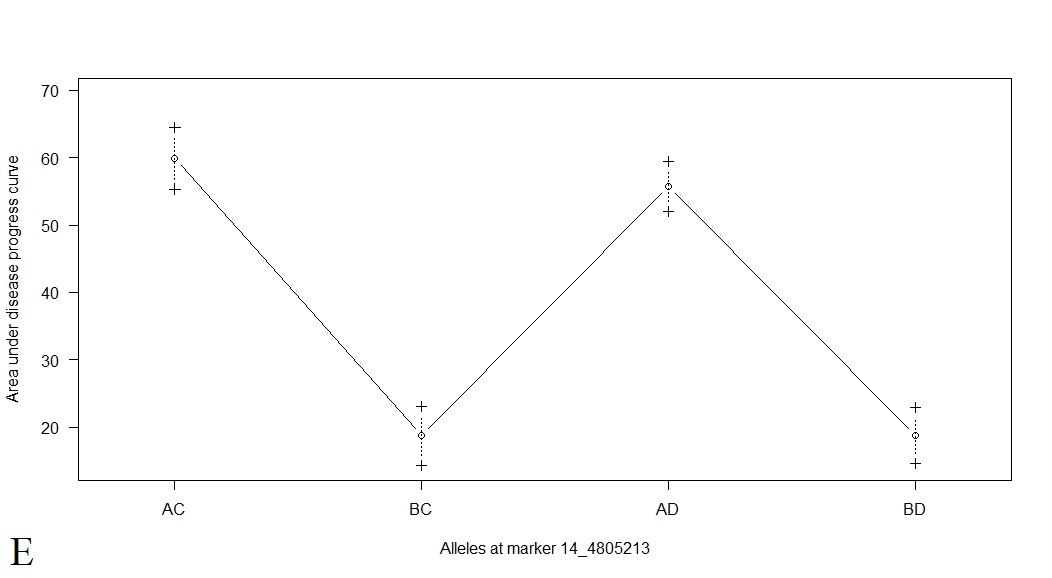

Supplement: Web_Material_uhac027 [file web_material_uhac027.zip › Supplementary Fig. 2E.jpg]

Area under disease progress curve

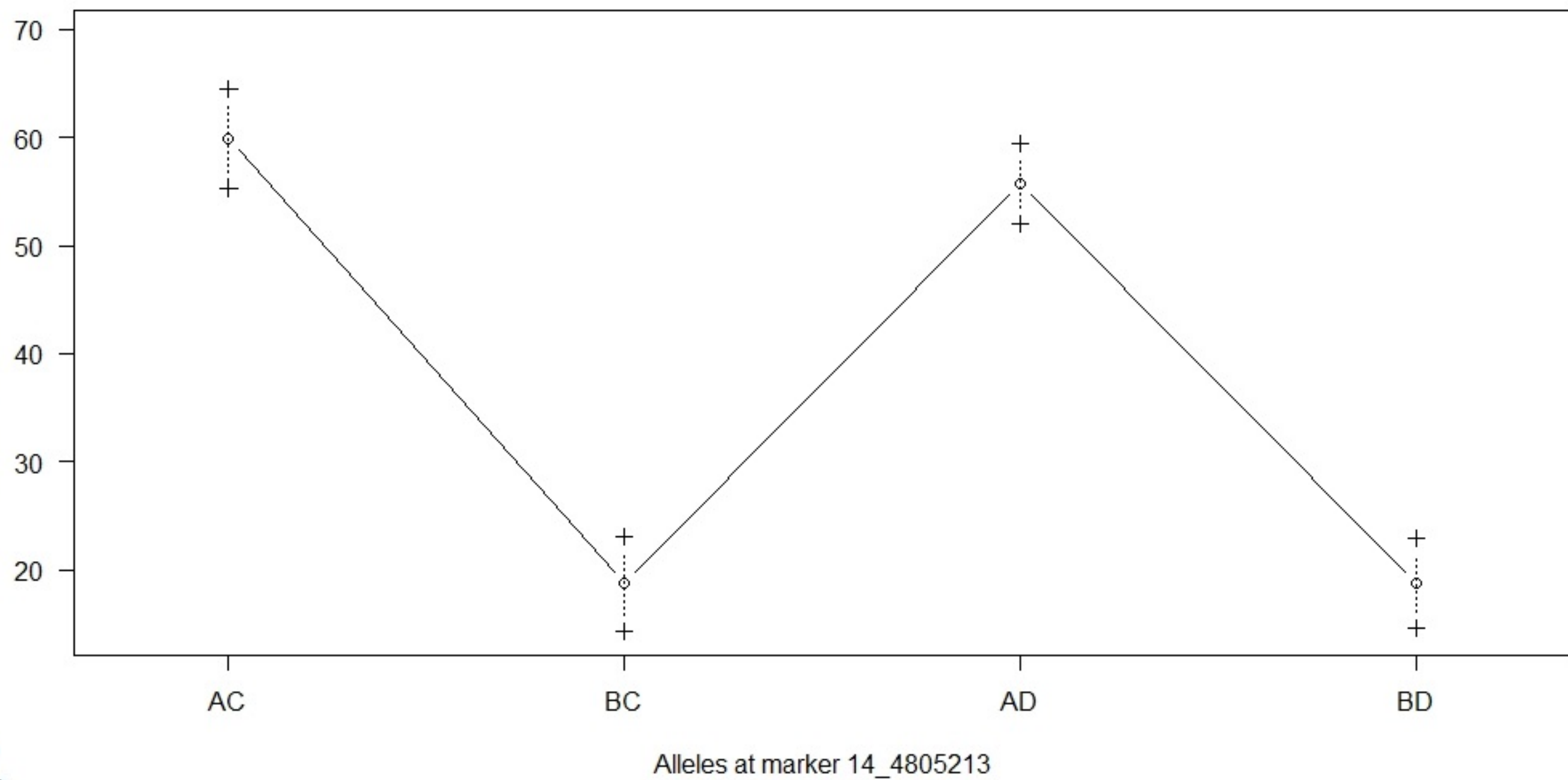

E

Supplement: Web_Material_uhac027 [file web_material_uhac027.zip › Supplementary Fig. 2E.pdf]

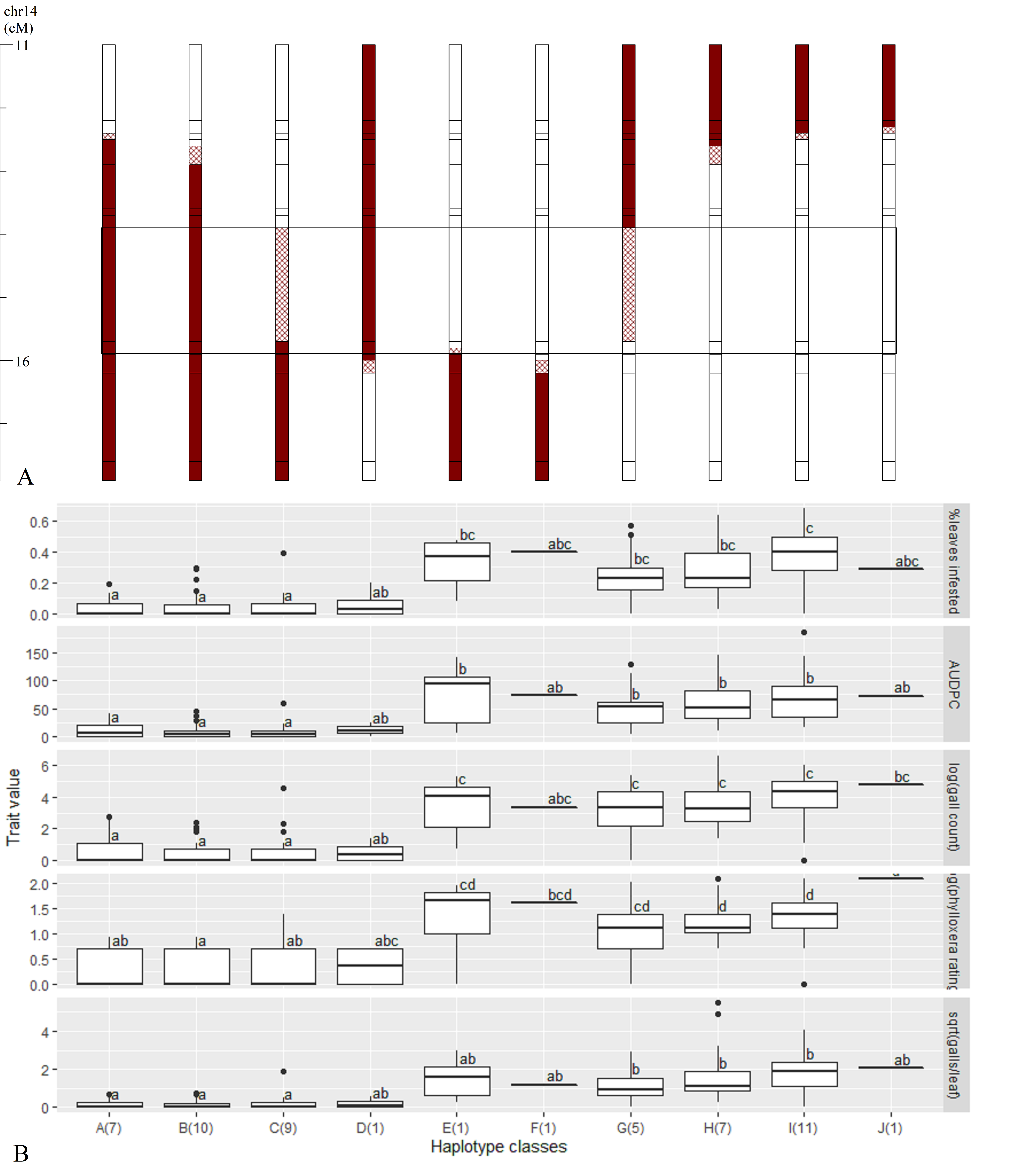

Supplement: Web_Material_uhac027 [file web_material_uhac027.zip › Supplementary Fig. 4.tif]

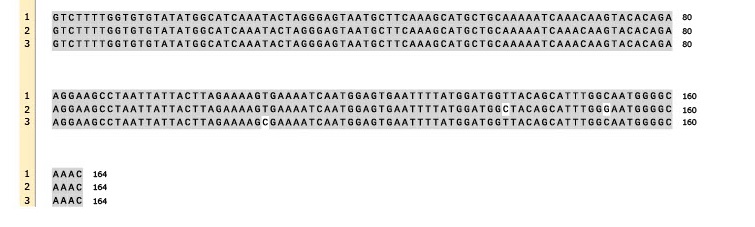

Supplement: Web_Material_uhac027 [file web_material_uhac027.zip › Supplementary Fig. 5.jpg]

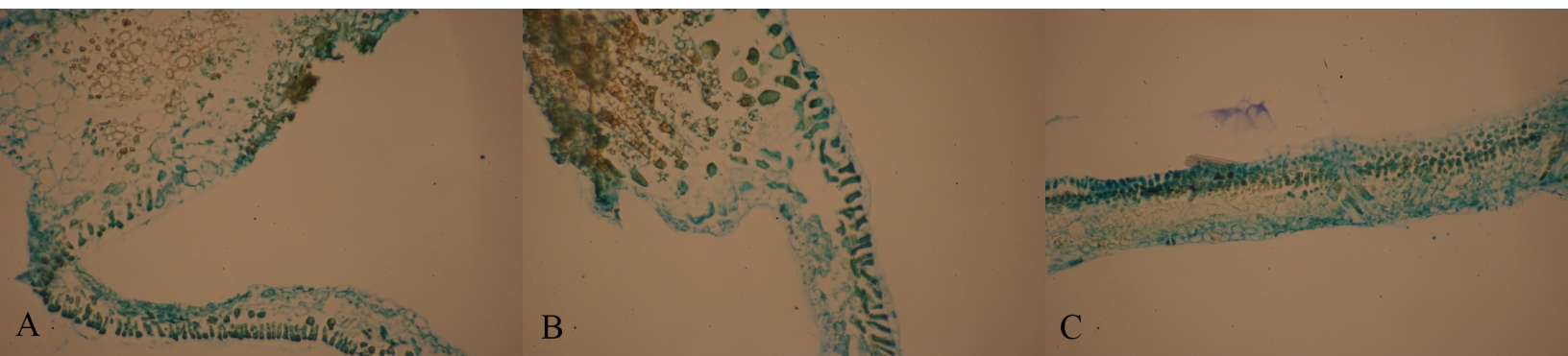

Supplement: Web_Material_uhac027 [file web_material_uhac027.zip › Supplementary Fig. 6.pdf]

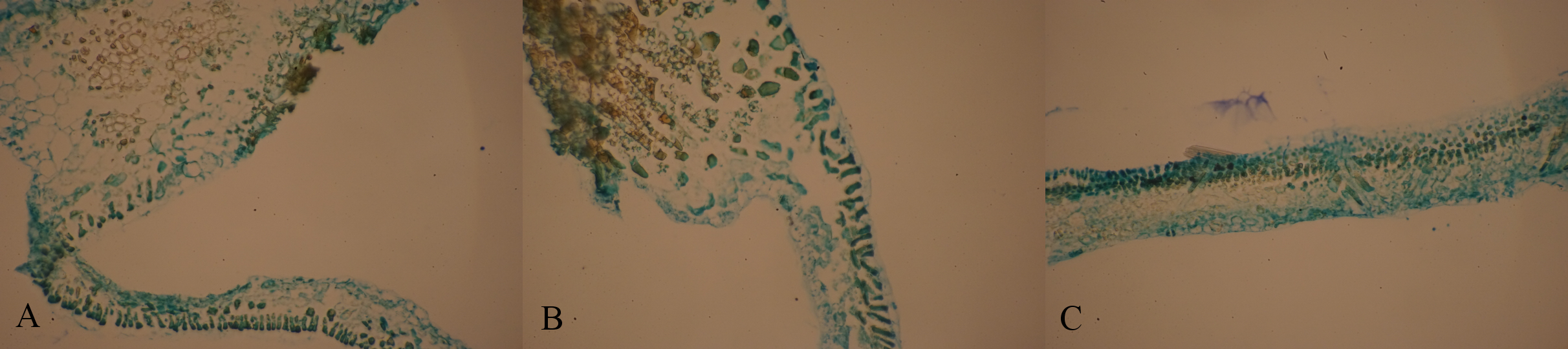

Supplement: Web_Material_uhac027 [file web_material_uhac027.zip › Supplementary Fig. 6.tif]
